# Dynamic Changes of Flavonoids Contents in the Different Parts of Rhizome of *Belamcanda chinensis* During the Thermal Drying Process

**DOI:** 10.3390/molecules190710440

**Published:** 2014-07-17

**Authors:** Yan Zhu, Bing-Qing Pu, Guo-Yong Xie, Mei Tian, Fang-Yun Xu, Min-Jian Qin

**Affiliations:** Department of Resources Science of Traditional Chinese Medicines, State Key Laboratory of Natural Medicines, China Pharmaceutical University, Nanjing 210009, China; E-Mails: cpuzy@126.com (Y.Z.); kypbq@163.com (B.-Q.P.); guoyongxie321@163.com (G.-Y.X.); ivytian11@hotmail.com (M.T.); xufangyun2010@163.com (F.-Y.X.)

**Keywords:** *Belamcanda chinensis*, thermal drying process, flavonoids, quantitation

## Abstract

The dried rhizome of *Belamcanda. chinensis* (L.) DC. is an important traditional Chinese medicine. Previous chemical and pharmacological investigations indicated that flavonoids may be responsible for the bioactivity of the herb. In this paper, the effects on the contents of twelve flavonoids in the three subunit parts of the rhizome of *B. chinensis* during the thermal drying process under treatment temperatures ranging from 40 °C to 120 °C at 10 °C intervals were investigated. The results showed that the content of most of the individual flavonoids except that of tectorigenin in the fresh eldest parts of the rhizome that originate directly from the seedling was higher than those of the other junior parts. The change trends of flavonoids contents were similar for three subunit parts of the rhizome during the drying process under the same treatment temperature. Most of the individual flavonoid contents in the rhizome increased in the early stages of the drying processes and decreased as the process was prolonged. The durations required to reaching the points of the maximal amounts of flavonoids revealed a significant negative correlation with the temperature. The variation of the content of mangiferin, iristectorigenin A, irigenin, irilone and dichotomitin was positively correlated with irisflorentin that is the chemical marker used for the quality control of this herb. Taking into account of the production effectiveness and flavonoid yields, the appropriate drying temperature for this herb was suggested to be 100 °C.

## 1. Introduction

*Belamcanda chinensis* (L.) DC. (Iridaceae) is a perennial herbaceous plant mainly distributed in China, Japan, Korea, India and eastern Russia. The dried rhizome of *B. chinensis* (Belamcandae Rhizoma, “Shegan” in Chinese) has been used as an important traditional Chinese herb medicine for thousands of years to cure pulmonary diseases, acute and chronic pharyngitis and asthma [1]. Previous chemical and pharmacological investigations indicated that flavonoids may be responsible for the bioactivity of Belamcandae Rhizoma [2,3,4,5,6,7]. Among them, tectoridin, tectorigenin, iridin, irigenin and irisflorentin were the most abundant bioactive constituents [8].

**Figure 1:**
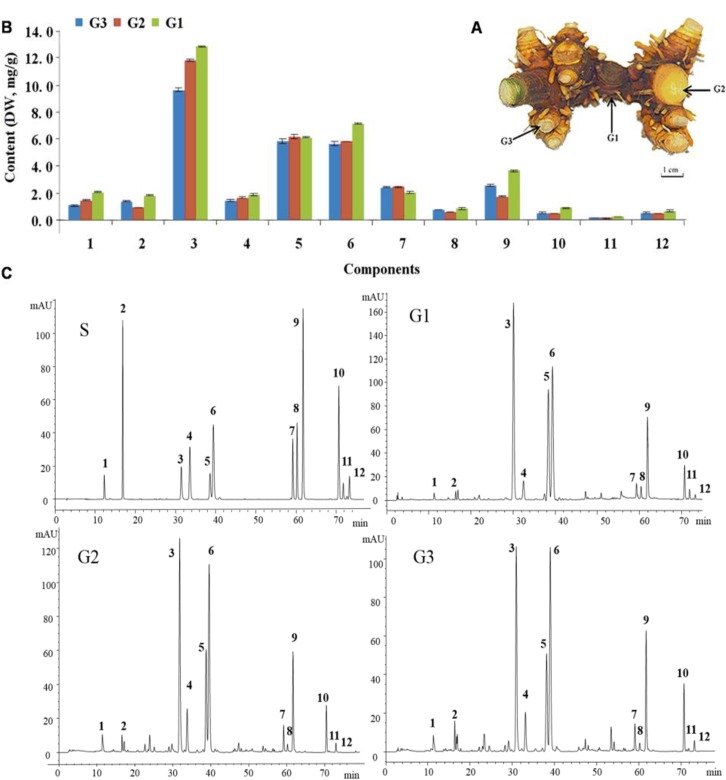
(**A**) The modules of the rhizome of *B. chinensis.* (**G1**–**G3** are the primary, second and third grade, respectively) (**B**) The comparison of components in three parts of fresh rhizome of *B. chinensis*. (**C**) Chromatograms of authentic standards and the extracts of different grades of Blamcandae Rhizoma Peaks: **1**, neomangiferin; **2**, mangiferin; **3**, tectoridin; **4**, iristectorin B; **5**, iristectorin A; **6**, iridin; **7**, tectorigenin; **8**, iristectorigenin A; **9**, irigenin; **10**, irisflorentin; **11**, irilone; **12**, dichotomitin.

At present, in the traditional medicinal raw materials markets in China, the major commodities of Belamcandae Rhizoma are the biennial rhizomes harvested from plantations. *B. chinensis* is a modular organism and its rhizome is composed of three levels of repeated, subunit parts (modules **G1**, **G2** and **G3** in Figure 1A) which are formed at different development stages of the plants. According to our previous biological research on *B. chinensis* [9], at the end of the first year of sowing, the young seedling of *B. chinensis* forms a main rhizome (the primary rhizome, grade I rhizome, **G1**), and 3~5 rhizomatous branches (the secondary rhizome, grade II rhizome, **G2**) on which 2~3 dormant buds initiate. In the second year, two or three of **G2** rhizomatous branches possessing a growth advantage produce three or four new rhizomatous branches (the third grade rhizome, grade III rhizome, **G3**). Up to now, comparisons of bioactive constituents among different subunit parts of the rhizome of *B. chinensis* have not been reported.

Quality control of the main bioactive components is important for the safety, efficacy and consistency for herbal medicines [10]. Generally, the components of herbal medicines may be influenced by the plant breed, organ specificity, stages of growth, cultivation parameters, harvesting times, processing, and storage conditions and so on [11,12,13,14,15,16,17]. To produce effective and constant herbal products, attention must be paid to these influencing factors.

Drying is a crucial and fundamental procedure in the post-harvest process which may affect the quality attributes (e.g., chemical compositions or content of the active principles and bioactivities) of medicinal herbs [18,19,20,21,22]. Conventionally, the rhizome of *B. chinensis* is processed through a natural sun drying process, however, takes a long time, more than one or two months, to reach the standard level of moisture (≤10.0%) documented in the Chinese Pharmacopoeia [1]. In the natural drying process of herbs, as the drying conditions are uncontrollable, this leads to the inability to guarantee the safety and consistency of active principle contents. Thermal drying is the most commonly employed commercial technique for drying herbs on an industrial scale [20]. However, so far, the influences of the thermal drying regime on the flavonoids contents of the rhizomes of *B. chinensis* remains to be clarified.

To provide basic information for the quality control and further optimization of the thermal drying process of *B. chinensis*, this paper reports in detail the effects of different treatment temperatures on the flavonoid contents of the herb during the thermal drying process. The comparisons of flavonoid contents among different growth and development level parts of the fresh rhizome were studied concurrently.

## 2. Results and Discussion

### 2.1. Dehydration Curves

**Figure 2:**
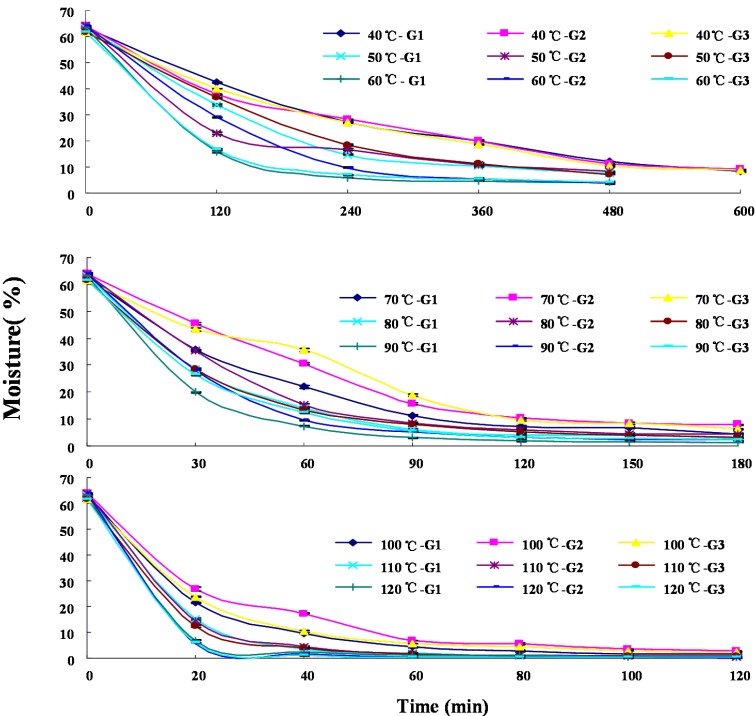
Dehydration curves of samples at different drying temperatures during the thermal drying process of the three grades parts of rhizomes of *B. chinensis*. **G1**, **G2** and **G3** are the primary, second and third grade parts of the rhizome, respectively.

Dehydration curves that indicated the changes of the moisture of the materials during the thermal drying process of the three grades of fresh rhizomes of *B. chinensis* at different temperatures are illustrated in Figure 2. The initial moisture contents of the three grades of rhizome of *B. chinensis* were 63.2% ± 0.2%, 64.0% ± 0.2% and 61.6% ± 0.6% (wet basis), respectively. As the temperature increased, the durations required reaching the desired standard moisture (≤10.0%) according to the Chinese Pharmacopoeia [1] from their initial values (~63%) of fresh materials were shortened significantly (Table 1) The drying duration reaching the desired moisture at 120 °C was only 20 min, while 10 h needed when dried at 40 °C. The results revealed a significant positive correlation between drying rate and treatment temperatures (*p* < 0.01).

**Table 1:** Efficiencies and levels of twelve analytes in dried samples with standard moisture. ^a^

G. ^b^	T ^c^ (°C)	Time (min)	Moist. ^d^	Contents of Analytes (mg/g, DW)											
				1	2	3	4	5	6	7	8	9	10	11	12
**G1**	40	600	8.13 ± 0.06	1.17 ± 0.10	1.23 ± 0.06	14.17 ± 0.04	1.83 ± 0.02	7.74 ± 0.15	7.21 ± 0.04	1.30 ± 0.09	0.60 ± 0.01	2.39 ± 0.03	0.49 ± 0.01	0.14 ± 0.02	1.05 ± 0.02
	50	420	8.41 ± 0.33	2.45 ± 0.05	1.65 ± 0.01	13.32 ± 0.01	1.89 ± 0.00	6.18 ± 0.19	7.19 ± 0.02	1.32 ± 0.00	0.76 ± 0.01	3.13 ± 0.00	0.90 ± 0.00	0.21 ± 0.01	0.92 ± 0.01
	60	180	8.53 ± 0.32	2.10 ± 0.06	1.92 ± 0.08	13.72 ± 0.08	2.01 ± 0.05	6.91 ± 0.07	8.35 ± 0.14	1.36 ± 0.09	0.86 ± 0.05	3.71 ± 0.23	1.10 ± 0.08	0.27 ± 0.01	1.00 ± 0.00
	70	120	7.35 ± 0.40	2.10 ± 0.23	1.65 ± 0.07	11.79 ± 0.13	1.73 ± 0.02	5.83 ± 0.03	7.04 ± 0.00	1.19 ± 0.09	0.70 ± 0.00	3.13 ± 0.08	0.93 ± 0.00	0.19 ± 0.02	0.81 ± 0.01
	80	90	6.06 ± 0.31	1.79 ± 0.03	2.09 ± 0.14	13.47 ± 0.03	2.06 ± 0.08	6.36 ± 0.04	7.72 ± 0.10	1.23 ± 0.05	0.87 ± 0.05	3.73 ± 0.22	1.28 ± 0.10	0.30 ± 0.00	1.16 ± 0.06
	90	60	7.33 ± 0.19	1.73 ± 0.12	1.59 ± 0.08	13.69 ± 0.26	2.01 ± 0.07	6.12 ± 0.07	7.48 ± 0.09	1.04 ± 0.03	0.79 ± 0.04	3.36 ± 0.06	1.06 ± 0.05	0.26 ± 0.00	0.91 ± 0.03
	100	40	9.69 ± 0.35	1.69 ± 0.03	1.74 ± 0.00	13.25 ± 0.03	2.07 ± 0.17	7.13 ± 0.13	8.30 ± 0.01	1.40 ± 0.06	0.97 ± 0.00	4.17 ± 0.01	1.38 ± 0.01	0.33 ± 0.01	1.25 ± 0.02
	110	40	3.57 ± 0.16	1.83 ± 0.01	1.64 ± 0.01	12.34 ± 0.00	1.90 ± 0.01	6.05 ± 0.04	7.94 ± 0.01	1.03 ± 0.04	0.79 ± 0.02	3.39 ± 0.06	1.00 ± 0.02	0.24 ± 0.00	0.91 ± 0.00
	120	20	7.00 ± 0.35	1.61 ± 0.07	1.85 ± 0.03	12.20 ± 0.04	1.90 ± 0.09	6.09 ± 0.03	7.04 ± 0.05	1.03 ± 0.03	0.87 ± 0.07	3.42 ± 0.05	0.91 ± 0.17	0.25 ± 0.04	0.71 ± 0.04
**G2**	40	600	9.03 ± 0.12	0.99 ± 0.00	1.11 ± 0.07	15.66 ± 0.27	1.81 ± 0.01	6.59 ± 0.11	5.97 ± 0.01	1.03 ± 0.02	0.61 ± 0.03	1.86 ± 0.00	0.40 ± 0.01	0.13 ± 0.01	0.67 ± 0.00
	50	420	8.40 ± 0.15	0.98 ± 0.01	1.46 ± 0.01	12.11 ± 0.18	1.66 ± 0.03	6.20 ± 0.06	6.65 ± 0.03	0.88 ± 0.03	0.63 ± 0.01	2.34 ± 0.06	0.67 ± 0.02	0.18 ± 0.01	0.55 ± 0.02
	60	240	9.62 ± 0.17	1.12 ± 0.00	1.25 ± 0.05	14.59 ± 0.29	1.84 ± 0.01	6.99 ± 0.12	7.32 ± 0.14	1.15 ± 0.01	0.70 ± 0.02	2.47 ± 0.06	0.64 ± 0.01	0.16 ± 0.01	0.73 ± 0.01
	70	150	8.39 ± 0.23	1.01 ± 0.01	1.28 ± 0.01	11.86 ± 0.27	1.59 ± 0.01	6.40 ± 0.13	6.69 ± 0.10	1.04 ± 0.02	0.73 ± 0.02	2.61 ± 0.06	0.64 ± 0.01	0.16 ± 0.00	0.62 ± 0.01
	80	90	8.51 ± 0.31	1.13 ± 0.04	1.62 ± 0.03	11.48 ± 0.32	1.73 ± 0.05	6.28 ± 0.17	7.07 ± 0.14	1.15 ± 0.02	0.83 ± 0.03	3.18 ± 0.02	0.88 ± 0.01	0.20 ± 0.01	0.96 ± 0.04
	90	60	9.53 ± 0.23	1.10 ± 0.05	1.22 ± 0.03	11.56 ± 0.12	1.62 ± 0.01	5.66 ± 0.03	6.25 ± 0.00	1.22 ± 0.01	0.83 ± 0.02	2.78 ± 0.04	0.73 ± 0.02	0.18 ± 0.01	0.73 ± 0.01
	100	60	6.78 ± 0.11	1.02 ± 0.02	1.56 ± 0.04	11.55 ± 0.14	1.48 ± 0.03	6.14 ± 0.16	6.44 ± 0.11	0.98 ± 0.01	0.85 ± 0.03	3.40 ± 0.06	1.02 ± 0.04	0.25 ± 0.01	0.80 ± 0.01
	110	40	4.41 ± 0.38	1.23 ± 0.01	1.54 ± 0.02	11.71 ± 0.34	1.62 ± 0.03	5.86 ± 0.10	6.17 ± 0.16	0.92 ± 0.01	0.68 ± 0.02	2.73 ± 0.02	0.76 ± 0.02	0.20 ± 0.01	0.74 ± 0.03
	120	20	5.53 ± 0.31	1.18 ± 0.03	1.03 ± 0.07	11.34 ± 0.29	1.55 ± 0.04	5.52 ± 0.13	6.89 ± 0.11	1.06 ± 0.03	0.78 ± 0.02	1.86 ± 0.04	0.77 ± 0.03	0.19 ± 0.01	0.56 ± 0.02
**G3**	40	600	8.57 ± 0.13	1.08 ± 0.01	1.53 ± 0.00	13.64 ± 0.01	1.75 ± 0.00	6.28 ± 0.03	5.81 ± 0.02	1.38 ± 0.11	0.69 ± 0.00	2.85 ± 0.07	0.50 ± 0.02	0.14 ± 0.00	0.49 ± 0.04
	50	420	7.57 ± 0.09	1.1 ± 0.05	1.9 ± 0.00	11.5 ± 0.00	1.7 ± 0.00	6.4 ± 0.00	6.4 ± 0.00	1.2 ± 0.00	0.8 ± 0.00	3.2 ± 0.01	0.6 ± 0.01	0.2 ± 0.00	0.6 ± 0.00
	60	240	7.01 ± 0.16	1.06 ± 0.00	1.96 ± 0.00	11.20 ± 0.00	1.70 ± 0.00	6.08 ± 0.00	6.04 ± 0.00	1.43 ± 0.00	0.83 ± 0.00	3.73 ± 0.00	0.71 ± 0.00	0.18 ± 0.00	0.74 ± 0.00
	70	120	9.51 ± 0.43	1.38 ± 0.01	1.68 ± 0.18	10.85 ± 0.10	1.44 ± 0.14	6.25 ± 0.06	5.58 ± 0.05	1.55 ± 0.19	0.88 ± 0.09	3.86 ± 0.05	0.59 ± 0.05	0.20 ± 0.00	0.71 ± 0.00
	80	90	8.16 ± 0.31	1.00 ± 0.05	1.61 ± 0.06	11.41 ± 0.50	1.58 ± 0.04	6.02 ± 0.23	5.65 ± 0.05	1.14 ± 0.06	0.78 ± 0.03	3.26 ± 0.01	0.55 ± 0.01	0.17 ± 0.00	0.62 ± 0.00
	90	90	5.44 ± 0.30	1.05 ± 0.01	1.82 ± 0.11	11.24 ± 0.03	1.79 ± 0.06	7.18 ± 0.02	6.86 ± 0.14	1.07 ± 0.08	0.81 ± 0.03	3.63 ± 0.29	0.69 ± 0.08	0.20 ± 0.03	0.59 ± 0.04
	100	60	5.62 ± 0.15	0.98 ± 0.03	1.56 ± 0.12	10.64 ± 0.08	1.57 ± 0.05	6.50 ± 0.04	5.96 ± 0.14	1.32 ± 0.03	0.90 ± 0.05	3.94 ± 0.25	0.72 ± 0.02	0.21 ± 0.00	0.87 ± 0.00
	110	40	4.00 ± 0.25	0.96 ± 0.03	1.82 ± 0.08	10.52 ± 0.19	1.59 ± 0.04	6.80 ± 0.15	6.89 ± 0.23	1.11 ± 0.07	0.87 ± 0.08	3.71 ± 0.03	0.79 ± 0.10	0.21 ± 0.01	0.93 ± 0.01
	120	20	5.99 ± 0.19	0.99 ± 0.04	1.49 ± 0.00	10.30 ± 0.44	1.52 ± 0.09	6.38 ± 0.19	6.15 ± 0.20	0.89 ± 0.06	0.71 ± 0.03	3.31 ± 0.11	0.67 ± 0.04	0.20 ± 0.00	0.68 ± 0.01

^a^ Results are mean values of triplicate assays; Content is the value of dry weight; ^b^ Grade; ^c^ Temperature; ^d^ Moisture (%). Numbering of the compounds is, **1**, neomangiferin; **2**, mangiferin; **3**, tectoridin; **4**, iristectorin B; **5**, iristectorin A; **6**, iridin; **7**, tectorigenin; **8**, iristectorigenin A; **9**, irigenin; **10**, irisflorentin; **11**, irilone; **12**, dichotomitin.

### 2.2. Determination of Analytes

In this research, our previous established HPLC-UV method for the simultaneous determination of the twelve analytes in Belamcandae Rhizoma was used [9], by which all twelve analytes (1~12) were well resolved with good peak symmetry (Figure 1C). This quantitative method was fully validated. The calibration curve, detection limit and quantification limit, and linear range for each analyte were provided in Supplementary Table S1. The recoveries of the twelve investigated components ranged from 97.97% to 104.08%. The intra- and inter-day precisions (RSD) were in the range of 0.92%~2.15% and 1.94%~3.54%, respectively. All the results revealed a high sensitivity and good linearity of the method. The results of determination of twelve flavonoids contents in three grades of rhizome of *B. chinensis* from different sampling points during drying process at different temperature were provided in Supplementary Tables S2–S4.

### 2.3. Differences of the Flavonoids Contents in Three Subunit Parts of Belamcandae Rhizoma

The main bioactivities of Belamcandae Rhizoma are ascribed to its abundant flavonoids, including isoflavonoid-*O*-glucosides, isoflavonoid aglycones and xanthones which contribute to estrogenic, antioxidative and anti-cancer effects [4,23,24,25]. The predominant isoflavonoid-*O*-glucosides in the Belamcandae Rhizoma include tectoridin (**3**), iristectorin A (**4**), iristectorin B (**5**) and iridin (**6**). The major flavonoid aglycones are tectorigenin (**7**), iristectorigenin A (**8**), irigenin (**9**), irisflorentin (**10**), irilone (**11**) and dichotomitin (**12**). The major xanthones in the rhizome are neomangiferin (**1**) and mangiferin (**2**), which are also flavonoid-glucosides. The results revealed that the contents of most of the ingredients except tectorigenin (**7**) in the fresh **G1** were significantly higher than those of the fresh **G2** and **G3** (Figure 1B). The content of tectoridin (**3**), the main ingredient in the fresh **G1** was 12.85 ± 0.06 mg/g DW, while the contents of those in the fresh **G2** and **G3** were 11.87 ± 0.07 mg/g DW and 9.65 ± 0.03 mg/g DW, respectively. The differences of the flavonoids contents between fresh **G2** and **G3** were also significant, except iridin (**6**), tectorigenin (**7**), irisflorentin (**10**) and dichotomitin (**12**). The contents of neomangiferin (**1**), tectoridin (**3**), iristectorin B (**4**), iristectorin A (**5**) in the fresh **G2** were slightly higher than those of fresh **G3**, while contents of mangiferin (**2**), iristectorigenin A (**8**), irigenin (**9**) and irilone (**11**) in the fresh **G2** were slightly lower than those in fresh **G3** (Figure 1B). The results indicated that the elder parts of the rhizome accumulated more amounts of flavonoid than the junior ones. It might be due to the characteristic chemical ingredients distribution in the different tissues of the rhizome. According to our preliminary study on histochemical analysis of the rhizome of *B. chinensis*, xanthones and isoflavonoid aglycones distribute mainly in the external cork cell layer of the rhizomes, while isoflavonoid-*O*-glucosides mainly locate in the internal parenchyma cells and vascular bundle tissue cells (unpublished data). When the rhizomes were separated into three subunit parts, the **G2** possessed the least proportion of external cork cells layer to internal tissues, which might explain that the contents of most isoflavonoid aglycones in the fresh **G2** were slightly lower than those in fresh **G3**. The similar phenomenon was also observed in the root of *Salvia miltiorrhiza* and the report indicated that the lipophilic diterpenoid quinonoes mainly distributed in the bark and increased with the cultivated years [26].

### 2.4. Dynamic Changes of Flavonoids in Thermal Drying Process

**Figure 3:**
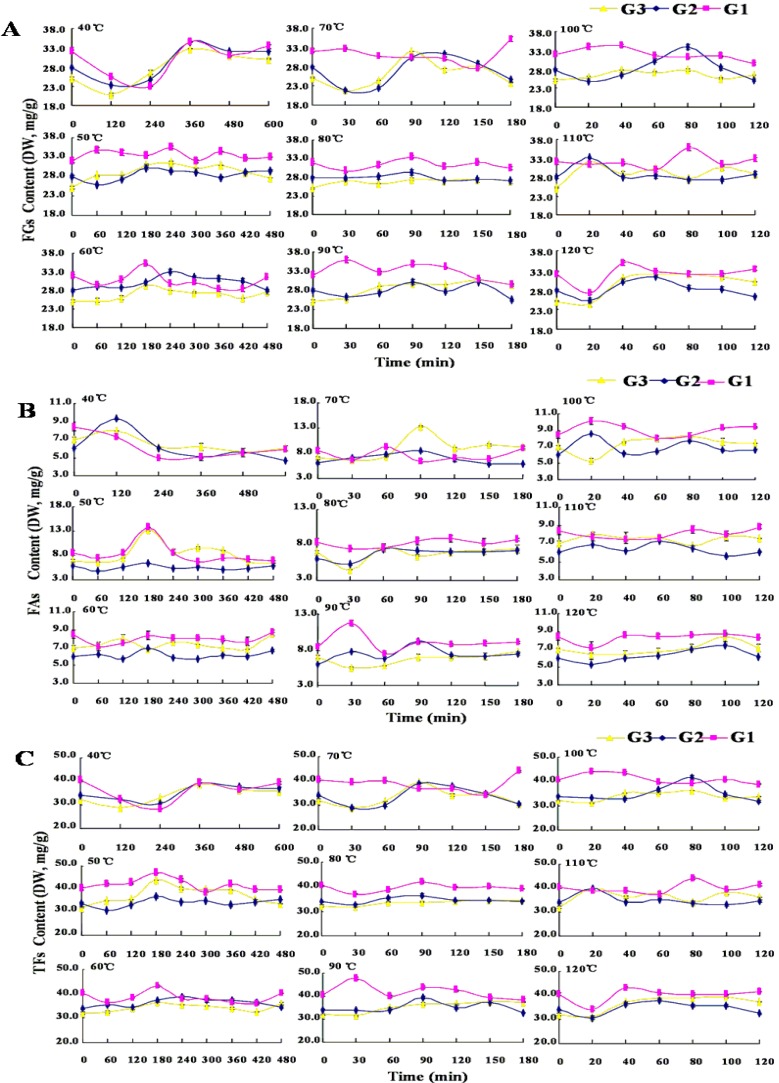
The contents of **FGs** (**A**), **FAs** (**B**) and **TFs** (**C**) during the drying process at different temperatures. **G1**, grade I rhizome; **G2**, grade II rhizome; **G3**, grade III rhizome.

During the thermal drying processes, the contents of most individual flavonoids and the total sum content of twelve flavonoids (**TFs**) in the rhizome of *B. chinensis* increased in the early stages of the thermal drying processes and decreased as the processes prolonged (Figure 3). The duration reaching the points of maximum flavonoids contents showed a negative correlation with treatment temperatures. The change trends of flavonoids contents in the three grades parts of the rhizome were similar during thermal drying process at the same treatment temperatures, whereas the durations when the flavonoids contents reached maximum values (apical values, *A*-values) and the rates of change at the same temperature for the three grades samples of the rhizome varied. The flavonoids contents of sufficiently dried samples (*S*-values) of each ingredient were lower than their *A*-values due to a longer drying process the materials underwent.

In the treatment sets of **G1**, the maximal *A*-values of the total contents of both flavonoid-glucosides **(FGs**) and **TFs** were at 90 °C for 30 min, which were 35.91 ± 0.61 mg/g DW and 47.64 ± 0.50 mg/g DW, respectively. While the maximal *A*-value of the total content of flavonoid aglycones (**FAs**), which were 13.74 ± 0.05 mg/g DW appeared at 50 °C for 180 min. Compared to those of fresh **G1**, the amounts of **FGs**, **FAs** and **TFs** were increased by 12.29%, 62.80% and 17.86%, respectively. In the treatment sets of **G2**, the maximal A-values of **FGs** and **FAs** were observed at 40 °C for 360 min and 120 min, respectively, 34.64 ± 0.57 mg/g DW and 9.71 ± 0.04 mg/g DW, respectively, The maximal *A*-values of **TFs** were at 100 °C for 80 min, which were 41.68 ± 0.82 mg/g DW. The amounts of **FGs**, **FAs** and **TFs** increased 23.71%, 61.56% and 22.59%, respectively, compared to those in fresh **G2**. In the treatment sets of **G3**, the maximal contents of **FAs** (13.35 ± 0.40 mg/g DW) and **TFs** (44.10 ± 0.08 mg/g DW) were achieved at 50 °C for 180 min, increased 90.99% and 37.34% respectively, compared to those in fresh **G3**, while the maximal *A*-values of **FGs (**32.74 ± 0.40 mg/g DW) was at 40 °C for 360 min with an increase of 30.33% to those of the fresh **G3** (Figure 3).

It is generally acknowledged that the contents of bioactive components in medicinal plants are accumulated in the pre-harvest and decrease in the post-harvest drying process along with the increase of temperature and the prolonging of treatment [27]. The results obtained from the present study showed that both low and high temperature drying treatments could lead to significant dynamic changes of the flavonoids contents in the rhizomes of *B. chinensis* during the thermal drying process. The change rates of flavonoids in the same grade of the rhizome during thermal drying process at the different temperatures were varied. At lower temperatures, the increase amounts of flavonoids at first in the early stages of thermal drying processes might be explained by the fact that the rhizome in drying process may be induced a series of stress resistance reactions. It is speculated that the fresh rhizome of *B. chinensis* remained physiologically active after harvested. The high temperature and dewatering stress on the plant might induce its physiological reaction and a lot of amount of reactive oxygen species (ROS) might be produced. Excess ROS might cause oxidative stress which could result in injury to plant at both the molecular and cellular level [28]. To avoid oxidative injury to the plant itself, excess ROS amounts might be particularly scavenged by antioxidant metabolites of plant [29]. During the thermal drying process of *B. chinensis* rhizome at low temperature, many biochemical reactions with the action of enzymes occur in the plant, which result in an increased amount of flavonoids that might be used as antioxidants [30,31,32], but this hypothesis needs further research to be confirmed. As the temperature increases, the enzymes in the plant will be deactivated and the cell walls will break down, and thereby the bonding forces between flavonoids and tissue matrix are weakened, leading to more flavonoids being released and higher contents of total flavonoids are detected [33]. Similar phenomena have been reported with some types of secondary metabolites in other medicinal or food materials such as *Salvia miltiorrhiza* (“Danshen” in chinese), North American Ginseng, maca (*Lepidium meyenii*) and tomato [22,33,34,35].

The change of the individual flavonoid content within the same grade of the rhizome was different during the thermal drying process at the different treatment temperatures. The fresh rhizome contained the highest amount of tectorigenin (**7**), which decreased during the drying process at all tested temperatures. It is possible that tectorigenin is more thermo-labile than other flavonoids, even at the low temperature. It is possible that tectorigenin was consumed first as an antioxidant during the drying process.

**Figure 4:**
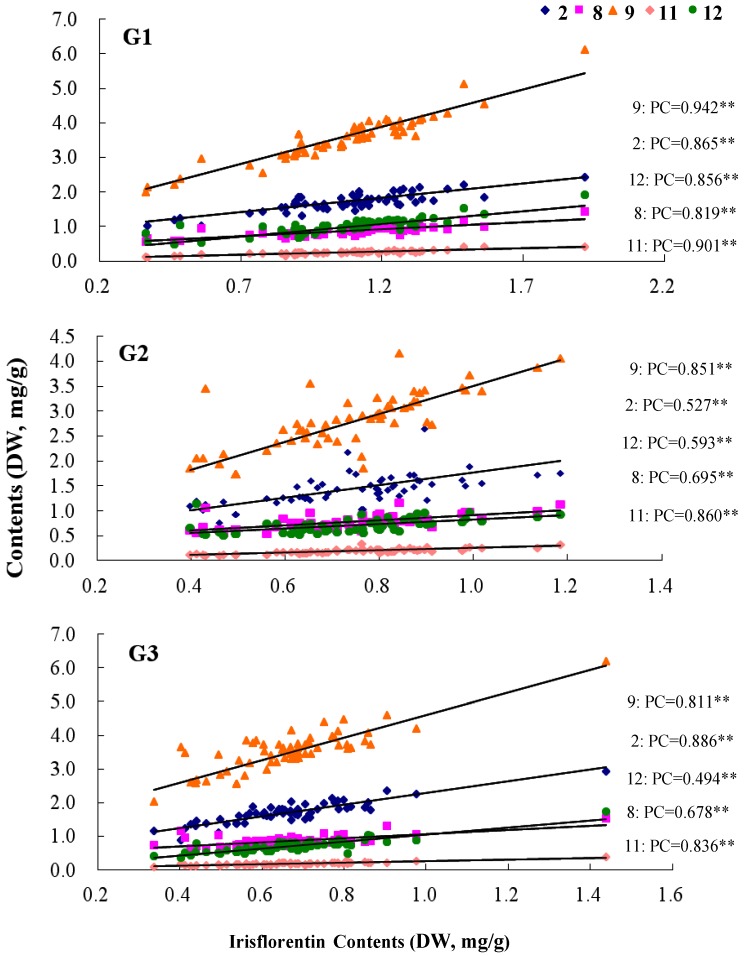
The correlations between irisflorentin and the compound **2**, **8**, **9**, **11** and **12**. **2**, mangiferin; **8**, iristectorigenin A; **9**, irigenin; **11**, irilone; **12**, dichotomitin. (PC is the Pearson Correlation between irisflorentin and the compound **2**, **8**, **9**, **11** and **12**, respectively).

Irisflorentin (**10**), a flavonoid aglycone, is the chemical marker used for the quality control of Belamcandae Rhizoma, and minimum content of this component in the commercial materials of this herb should be no less than 0.10% as documented in the Chinese Pharmacopoeia [1]. The variation of the contents of mangiferin (**2**), iristectorigenin A (**8**), irigenin (**9**), irilone (**11**) and dichotomitin (**12**), were positively correlated with irisflorentin (**10**) (*p* < 0.01) (Figure 4**)**. Interestingly, all of those flavonoids distribute mainly in the external cork cells layer of the rhizomes.

Although the amounts of the flavonoids in the rhizome of *B. chinensis* were increased in the early stages during thermal drying process, the efficiencies of thermal drying process under different treatment temperatures were varied. Table 1 showed the contents of the 12 flavonoids in the three grades of the rhizome at the sampling point when the moisture met the standard (≤10.0%).

In order to clarify the efficiency of thermal drying process for three grades of *B. chinensis*, the increase rate of flavonoid content was calculated with the following equation:

**IF** = (**S** − **F**)/**F** × 100% (1)

Where **IF** is the increase rate of flavonoid content (%), **S** is the flavonoid content in the three grades of the rhizome at the sampling point when the moisture met the standard (≤10.0%) (DW, mg/g) and **F** is the flavonoid content in the fresh rhizome (DW, mg/g). When IF value is positive indicate increasing the amount of flavonoid, and negative value indicates decreasing the amount of flavonoid. The **IF** values obtained for the three grades samples of the rhizome at different treatment temperatures were shown in Table 2. The **IF** values of **FGs**, **FAs** and **TFs** from each grade treated at 100 °C showed positive values, which were 6.88%, 12.53% and 8.06% for **G1**, 8.42%, 6.66% and 8.11% for **G2**, and 8.30%, 13.90% and 9.52% for **G3**, respectively. While all **IF** values of **FGs**, **FAs** and **TFs** from each grade treated at 120 °C were negative indicated that the flavonoids contents were all decreased at this treatment temperature. Most of **IF** values of **FGs** were positive ones except those of **G1** sets at 70 °C, 110 °C and 120 °C treatment, **G2** sets at 90 °C and 120 °C treatment. In contrast, most of **IF** values of **FAs** for **G1** sets were negative except those of 80 °C and 100 °C treatment. As to **IF** values of **FAs** for **G2** sets, those of low temperature (40 °C, 50 °C, 60 °C and 70 °C) treatment were negative while those of high temperature (80 °C, 90 °C, 100 °C and 110 °C) treatment were positive. **IF** values of **FAs** for **G3** sets, those of treatment at 40 °C, 50 °C, 80 °C and 120 °C were negative, while those of treatment at 60 °C, 90 °C, 100 °C and 110 °C were positive. Most of **IF** values of **TFs** for **G1** were negative ones except those of 60 °C, 80 °C and 100 °C treatment. All of **IF** values of **TFs** for **G2** and **G3** sets except that of 120 °C treatment were positive. This result implicated that the optimal drying temperature for the rhizomes of *B. chinensis* should be 60 °C, 80 °C and 100 °C. When the production effectiveness is taken into consideration, the most appropriate temperature for *B. chinensis* drying process is suggested to be 100 °C.

**Table 2:** The rate of increase of flavonoid contents when the moisture reached the standard.

	CF ^a^	40 °C	50 °C	60 °C	70 °C	80 °C	90 °C	100 °C	110 °C	120 °C
**G1**	**FGs** (%)	4.28	2.16	9.47	−5.75	4.68	1.98	6.88	−0.85	−14.85
	**FAs** (%)	−29.39	−14.24	−1.59	−17.68	1.46	−12.14	12.53	−12.84	−14.79
	**TFs** (%)	−2.75	−1.26	7.16	−8.24	4.01	−0.97	8.06	−3.36	−14.84
**G2**	**FGs** (%)	14.75	3.81	18.28	2.93	4.69	−2.07	8.42	0.47	−9.63
	**FAs** (%)	−21.90	−12.39	−2.60	−3.41	19.83	12.26	6.66	2.93	−13.17
	**TFs** (%)	8.28	0.94	14.59	1.81	7.36	0.46	8.11	0.91	−10.25
**G3**	**FGs** (%)	19.79	15.08	11.67	8.21	8.59	19.21	8.30	13.78	−3.11
	**FAs** (%)	−13.56	−5.48	8.88	27.34	−6.80	0.02	13.90	8.90	−7.65
	**TFs** (%)	12.54	10.61	11.06	6.70	5.24	15.03	9.52	12.71	−4.10

^a^ **IF** is the increase rate of flavonoid content (%).

## 3. Experimental Section

### 3.1. Plant Materials

Fresh two-year-old rhizomes of *B. chinensis* were collected from Tuanfeng County, Hubei Province in China, during the harvest season in October 2012 and were authenticated by one of the authors, Dr Minjian Qin. The voucher specimens (CMR00001MT017) were deposited in the Department of Resources Science of Traditional Chinese Medicines, China Pharmaceutical University, Nanjing, China. The fresh rhizomes were transported to the laboratory within two days and the soil was removed with a dry towel. Then the rhizome samples were immediately separated into three parts, namely the primary grade (grade I rhizome, **G1**), the second grade (grade II rhizome, **G2**) and the third grade (grade III rhizome, **G3**) as shown in Figure 1A, according to the levels of growth and development as described above. The raw materials were sliced into 2~3 mm pieces. The sliced materials were selected for uniform size, mixed well and stored at −20 °C until assay.

### 3.2. Drying Process

The sliced fresh materials were dried at different temperatures ranging from 40 °C to 120 °C in 10 °C intervals using a thermostatic oven. The sampling interval and frequency at each temperature were determined according to the difference of drying efficiency, specifically, 2 h intervals in total 10 h for 40 °C; 1 h intervals in total 8 h for 50 °C and 60 °C; 30 min intervals in total 3 h, for 70 °C, 80 °C and 90 °C; 20 min intervals in total 2 h for 100 °C, 110 °C and 120 °C. The sliced raw materials (~10.0 g) were weighted and medially placed on a watch glass (9.0 cm in diameter). Prior to put into the materials, the oven was preheated to the set temperature. At each sampling point, two batches (one for moisture determination, the other for contents determination) with each on three watch glasses, were randomly taken from the oven during the drying process. The processed materials were cooled to the room temperature in a desiccator, then grounded and sieved with 80 mesh sieve and weighed accurately.

### 3.3. Determination of Moisture

The results of the pre-experiment suggested that there was no significant difference between the drying method recommended in the Chinese Pharmacopoeia [1] and the method using a digital moisture analyzer. Detailed data are shown in Supplementary Material Table S5. The latter method could solve some problems such as a long time drying and tedious operation. The moisture of samples from different sampling points was determined in triplicate by a digital moisture analyzer (Satorius, MA35, Gottingen, Germany) at 105 °C.

### 3.4. Chemicals and Reagents

Reference compounds including neomangiferin (**1**), mangiferin (**2**), tectoridin (**3**), iristectorin B (**4**), iristectorin A (**5**), iridin (**6**), tectorigenin (**7**), iristectorigenin A (**8**), irigenin (**9**), irisflorentin (**10**), irilone (**11**) and dichotomitin (**12**) were isolated and purified from the dried rhizomes of *B. chinensis* (L.) DC. or *Iris tectorum* Maxim. in our laboratory [5,6,36]. Their structures were identified by UV, IR, MS, ^1^H-NMR and ^13^C-NMR, and the purity of each compound was determined to be over 98% by an HPLC-UV method. HPLC grade acetonitrile (Merck, Darmstadt, Germany) was used for HPLC analysis. Ultra-pure water was purified from a ULUP-II-20T purification system (ULUP, Nanjing, China); analytical grade acetic acid (Nanjing Reagent, Jiangsu, China) and HPLC grade methanol (Hanbang, Jiangsu, China) were used for sample preparation.

### 3.5. Sample Preparation

The dry powders (~0.10 g) of Belamcandae Rhizoma were extracted twice with 75% methanol (20 mL) in an ultrasonic bath at room temperature for 30 min. The extracted solution was centrifuged at 3,000 rpm for 10 min, then combined and diluted with 75% methanol to 25 mL. The solution was filtered through a 0.45 µm membrane filter before HPLC analysis. Triplicate samples for each sampling point were analyzed.

### 3.6. Chromatographic Conditions

An Agilent 1100 Series HPLC instrument equipped with a UV detector, a quaternary pump, a column heater-cooler (Agilent Corporation, Palo Alto, CA, USA), a vacuum degasser and a 20 µL sample loop manual injector was used for sample analysis. The column configuration was an Agilent Zorbax SB-C_18_ column (250 mm × 4.6 mm, 5 µm) with an Alltech Associates C_18_ guard column (7.5 mm × 4.6 mm, 5 µm). The column temperature was maintained at 30 °C. The mobile phase consisted of solvent A (water-acetic acid (100:0.4, v/v)) and B (acetonitrile). The gradient elution program was used as follows: 8%–17% B in 0–15 min, 17%–20% B in 15–35 min, 20% B in 35–40 min, 20%–26% B in 40–65 min and 40%–50% B in 65–75 min. The flow rate was 1.0 mL/min. The injection volume was 20 µL. The detection wavelength was set at 269 nm for all the tested compounds.

### 3.7. Calibration and Method Validation

Known amounts of standards were dissolved and diluted with methanol to provide a series of standard solutions. The mixed standard stock solution containing 12 reference compounds were serially diluted for the construction of calibration curves at six concentrations. The limits of detection (LOD) and quantification (LOQ) were determined at a signal-to-noise (S/N) ration of 3 and 10, respectively.

The intra-day variability was performed six times within one day, and the inter-day variability was obtained from nine determinations in three consecutive days (three determinations per day). The recovery tests were performed by spiking a known amount of 12 standards into a Belamcandae Rhizoma sample and extracting as described above. All standard solutions of various concentrations were stored at 4 °C until assay. Each test was analyzed in triplicate.

### 3.8. Statistical Analysis

All data were the mean values from three samples in each sampling point and statistically analyzed by SPSS version 16.0 for Windows (SPSS Inc., Chicago, IL, USA). The least significant differences (LSD; *p* = 0.05), one way analysis of variance (ANOVA) and the bivariate correlation were calculated from each analysis.

## 4. Conclusions

This study revealed the differences of the contents of main flavonoids among the three subunit levels of the fresh rhizomes. The results indicated that the elder parts of the fresh rhizome contained higher amounts of flavonoids than the junior ones. The dynamic variation patterns of the amounts of flavonoids in the rhizome during the thermal drying process were obtained. Most of individual flavonoid contents in the rhizome increased in the early stages of the drying processes and decreased as the processes were prolonged. It is speculated that rhizomes of *B. chinensis* remained physiologically active after harvest and the stress resistance mechanisms of plants might be involved in the early stage of the herb drying process. The reasons for the flavonoid content variation in the rhizomeof *B. chinensis* during thermal drying processing needs to be further studied. Taking into account production effectiveness and flavonoid yields, the appropriate drying temperature for this herb was suggested to be 100 °C. These findings should facilitate the production of herbal products of rhizomes of *B. chinensis* with consistency and maximum flavonoid content.

## Supplementary Materials

Supplementary Materials can be accessed at: http://www.mdpi.com/1420-3049/19/7/10440/s1.
