# Supplementary material for: Dynamic Changes of Flavonoids Contents in the Different Parts of Rhizome of Belamcanda chinensis During the Thermal Drying Process
Source: Molecules. 2014 Jul 17;19(7):10440–54. doi: 10.3390/molecules190710440 (PMC6271185; doi:10.3390/molecules190710440)
Supplement: Supplementary file 1 [file molecules-19-10440-s001.pdf]

Supplementary Files

Table S1. Calibration curves, detection limits and quantification limits for the twelve investigated components (n = 6) of *B. chinensis*.

Analyte	Calibration Curve	R^2 (n = 6)	Linear Range (µg/mL)
Neomangiferin	$y = 28.17x + 2.68$	0.9999	0.27–55.00
Mangiferin	$y = 20.76x - 6.51$	0.9990	0.35–70.40
Tectoridin	$y = 94.19x - 37.26$	0.9998	3.86–154.50
Iristectorin B	$y = 82.51x - 10.64$	0.9999	1.65–66.00
Iristectorin A	$y = 49.80x - 43.19$	0.9992	1.10–110.00
Iridin	$y = 60.23x - 11.30$	0.9999	1.06–159.00
Tectorigenin	$y = 95.28x+25.32$	0.9999	0.27–108.50
Iristectorigenin A	$y = 56.82x - 4.84$	0.9999	0.16–16.20
Irigenin	$y = 109.93x + 5.03$	0.9998	0.83–41.60
Irisflorentin	$y = 123.90x - 0.19$	0.9993	0.23–11.70
Irilone	$y = 146.93x - 2.92$	0.9992	0.11–4.40
Dictomitin	$y = 18.20x - 1.47$	0.9991	0.59–17.70

y, peak area; *x*, the concentration of each reference compound (µg/mL).

Table S2-1. The data of rhizome of *B. chinensis* in **G1** from different sampling points at 40 ℃. [a]

T. [b]	Analytes	Drying Time (min)					
		**0**	**120**	**240**	**360**	**480**	**600**
	**Moist.** [c]	63.24 ± 0.21	42.31 ± 0.23	27.25 ± 0.06	20.14 ± 0.10	12.04 ± 0.13	8.13 ± 0.06
	**1** [d]	2.10 ± 0.07	0.95 ± 0.00	0.50 ± 0.11	1.37 ± 0.00	1.11 ± 0.07	1.17 ± 0.10
	**2** [d]	1.84 ± 0.03	1.02 ± 0.01	0.77 ± 0.06	1.20 ± 0.08	1.03 ± 0.05	1.23 ± 0.06
	**3** [d]	12.85 ± 0.06	11.12 ± 0.03	10.55 ± 0.03	15.58 ± 0.13	14.36 ± 0.13	14.17 ± 0.04
	**4** [d]	1.89 ± 0.12	1.41 ± 0.04	1.19 ± 0.04	2.00 ± 0.08	1.72 ± 0.02	1.83 ±0.02
	**5** [d]	6.14 ± 0.03	5.42 ± 0.02	4.72 ± 0.07	6.94 ± 0.10	6.59 ± 0.01	7.74 ± 0.15
**40 ℃**	**6** [d]	7.16 ± 0.03	5.33 ± 0.26	5.25 ± 0.01	7.41 ± 0.17	6.34 ± 0.08	7.21 ± 0.04
	**7** [d]	2.09 ± 0.07	2.05 ± 0.12	1.15 ± 0.04	1.20 ± 0.01	1.63 ± 0.13	1.30 ± 0.09
	**8** [d]	0.85 ± 0.07	0.94 ± 0.01	0.55 ± 0.02	0.58 ± 0.01	0.65 ± 0.08	0.60 ± 0.01
	**9** [d]	3.66 ± 0.03	2.97 ± 0.05	1.99 ± 0.02	2.20 ± 0.05	2.14 ± 0.06	2.39 ± 0.03
	**10** [d]	0.91 ± 0.07	0.56 ± 0.00	0.36 ± 0.04	0.46 ± 0.01	0.37 ± 0.04	0.49 ± 0.01
	**11** [d]	0.24 ± 0.01	0.19 ± 0.00	0.12 ± 0.02	0.14 ± 0.01	0.10 ± 0.02	0.14 ± 0.02
	**12** [d]	0.69 ± 0.05	0.54 ± 0.03	0.80 ± 0.00	0.50 ± 0.04	0.57 ± 0.01	1.05 ± 0.02

[a] Results are presented as means ± sd (n = 3), Content is the value of dry weight; [b] Temperature; [c] Moisture (%); [d] units: mg/g. Numbering of the compounds is, **1**, neomangiferin; **2**, mangiferin; **3**, tectoridin; **4**, iristectorin B; **5**, iristectorin A; **6**, iridin; **7**, tectorigenin; **8**, iristectorigenin A; **9**, irigenin; **10**, irisflorentin; **11**, irilone; **12**, dichotomitin.

Table S2-2. The data of rhizome of *B. chinensis* in **G1** from different sampling points at 50 ℃ and 60 ℃. [a]

T. [b]	Analytes	Drying Time (min)								
		0	60	120	180	240	300	360	420	480
50 ℃	Moist. [c]	63.24 ± 0.21	47.10 ± 0.39	33.82 ± 0.28	24.66 ± 0.39	14.71 ± 0.34	11.39 ± 0.53	10.25 ± 0.05	8.41 ± 0.33	7.68 ± 0.39
	1 [d]	2.10 ± 0.07	2.07 ± 0.04	2.15 ± 0.11	2.17 ± 0.00	1.97 ± 0.07	1.77 ± 0.10	2.71 ± 0.00	2.45 ± 0.00	2.31 ± 0.02
	2 [d]	1.84 ± 0.03	1.63 ± 0.01	1.77 ± 0.08	2.42 ± 0.02	1.66 ± 0.10	1.44 ± 0.01	1.72 ± 0.04	1.65 ± 0.01	1.56 ± 0.03
	3 [d]	12.85 ± 0.06	15.86 ± 0.05	14.65 ± 0.04	13.84 ± 0.00	14.98 ± 0.06	12.94 ± 0.16	13.40 ± 0.01	13.32 ± 0.01	13.36 ± 0.01
	4 [d]	1.89 ± 0.12	2.05 ± 0.00	2.08 ± 0.17	1.80 ± 0.00	2.21 ± 0.11	2.01 ± 0.10	1.97 ± 0.09	1.89 ± 0.01	1.86 ± 0.02
	5 [d]	6.14 ± 0.03	6.26 ± 0.35	6.15 ± 0.10	6.04 ± 0.00	6.74 ± 0.08	6.30 ± 0.08	6.84 ± 0.01	6.18 ± 0.12	6.35 ± 0.01
	6 [d]	7.16 ± 0.03	6.91 ± 0.25	7.28 ± 0.11	7.12 ± 0.00	7.94 ± 0.02	7.55 ± 0.25	7.87 ± 0.03	7.19 ± 0.08	7.58 ± 0.02
	7 [d]	2.09 ± 0.07	1.54 ± 0.01	1.54 ± 0.17	1.92 ± 0.22	1.60 ± 0.03	1.41 ± 0.11	1.39 ± 0.00	1.32 ± 0.01	1.31 ± 0.01
	8 [d]	0.85 ± 0.07	0.83 ± 0.14	0.91 ± 0.09	1.44 ± 0.02	0.87 ± 0.17	0.80 ± 0.34	0.83 ± 0.01	0.76 ± 0.02	0.73 ± 0.00
	9 [d]	3.66 ± 0.03	3.10 ± 0.05	3.51 ± 0.29	6.11 ± 0.10	3.61 ± 0.01	2.54 ± 0.05	3.11 ± 0.02	3.13 ± 0.01	3.05 ± 0.02
	10 [d]	0.91 ± 0.07	0.89 ± 0.04	1.11 ± 0.08	1.92 ± 0.03	1.07 ± 0.08	0.78 ± 0.03	0.90 ± 0.00	0.90 ± 0.01	0.85 ± 0.01
	11 [d]	0.24 ± 0.01	0.23 ± 0.05	0.25 ± 0.01	0.42 ± 0.04	0.26 ± 0.05	0.23 ± 0.06	0.24 ± 0.00	0.21 ± 0.01	0.21 ± 0.01
	12 [d]	0.69 ± 0.05	0.90 ± 0.09	1.16 ± 0.04	1.92 ± 0.01	1.08 ± 0.08	1.00 ± 0.17	1.05 ± 0.00	0.92 ± 0.02	0.90 ± 0.01
60 ℃	Moist. [c]	63.24 ± 0.21	34.52 ± 0.46	16.00 ± 0.55	8.53 ± 0.32	5.85 ± 0.18	6.20 ± 0.20	4.63 ± 0.25	4.23 ± 0.58	4.08 ± 0.20
	1 [d]	2.10 ± 0.07	1.49 ± 0.05	2.04 ± 0.00	2.10 ± 0.01	1.88 ± 0.05	1.55 ± 0.00	1.65 ± 0.03	1.50 ± 0.08	1.57 ± 0.01
	2 [d]	1.84 ± 0.03	1.62 ± 0.02	1.56 ± 0.02	1.92 ± 0.01	1.65 ± 0.01	1.93 ± 0.11	1.65 ± 0.05	1.53 ± 0.05	2.01 ± 0.01
	3 [d]	12.85 ± 0.06	12.55 ± 0.10	12.58 ± 0.00	13.72 ± 0.10	11.62 ± 0.03	12.09 ± 0.09	11.65 ± 0.03	11.46 ± 0.05	12.92 ± 0.07
	4 [d]	1.89 ± 0.12	1.71 ± 0.03	1.75 ± 0.00	2.01 ± 0.01	1.70 ± 0.00	1.70 ± 0.19	1.69 ± 0.01	1.70 ± 0.04	1.91 ± 0.01
	5 [d]	6.14 ± 0.03	5.67 ± 0.05	5.92 ± 0.10	6.91 ± 0.04	5.78 ± 0.02	5.70 ± 0.02	5.29 ± 0.04	5.54 ± 0.00	6.03 ± 0.03
	6 [d]	7.16 ± 0.03	6.29 ± 0.04	7.06 ± 0.10	8.35 ± 0.02	7.14 ± 0.02	7.15 ± 0.19	6.51 ± 0.06	6.50 ± 0.04	7.11 ± 0.04
	7 [d]	2.09 ± 0.07	1.19 ± 0.04	1.33 ± 0.08	1.36 ± 0.08	1.36 ± 0.01	1.23 ± 0.05	1.37 ± 0.01	1.36 ± 0.04	1.53 ± 0.01
	8 [d]	0.85 ± 0.07	0.77 ± 0.02	0.75 ± 0.04	0.86 ± 0.01	0.80 ± 0.00	0.83 ± 0.06	0.88 ± 0.01	0.81 ± 0.06	0.95 ± 0.00
	9 [d]	3.66 ± 0.03	3.06 ± 0.09	3.33 ± 0.04	3.71 ± 0.07	3.56 ± 0.01	3.58 ± 0.07	3.41 ± 0.05	3.28 ± 0.02	3.85 ± 0.02
	10 [d]	0.91 ± 0.07	0.96 ± 0.01	0.97 ± 0.01	1.10 ± 0.05	1.13 ± 0.01	1.12 ± 0.06	1.06 ± 0.07	0.99 ± 0.02	1.12 ± 0.01
	11 [d]	0.24 ± 0.01	0.25 ± 0.00	0.22 ± 0.04	0.27 ± 0.01	0.21 ± 0.05	0.24 ± 0.06	0.22 ± 0.04	0.22 ± 0.00	0.28 ± 0.00
	12 [d]	0.69 ± 0.05	0.78 ± 0.01	0.88 ± 0.05	1.00 ± 0.00	0.95 ± 0.00	0.97 ± 0.10	0.95 ± 0.03	1.01 ± 0.03	0.95 ± 0.00

[a] Results are presented as means ± sd (n = 3), Content is the value of dry weight; [b] Temperature; [c] Moisture (%); [d] units: mg/g. Numbering of the compounds is the same as Table S2-1.

Table S2-3. The data of rhizome of *B. chinensis* in **G1** from different sampling points at 70 ℃, 80 ℃ and 90 ℃. [a]

T. [b]	Analytes	Drying Time (min)						
		0	30	60	90	120	150	180
70 ℃	**Moist.** [c]	63.24 ±0.21	35.63 ±0.24	21.95 ±0.44	11.39 ±0.11	7.35 ±0.33	6.86 ±0.40	4.45 ±0.10
	**1** [d]	2.10 ±0.07	1.77 ±0.19	2.10 ±0.09	1.79 ±0.30	2.10 ±0.09	2.06 ±0.03	1.89 ±0.12
	**2** [d]	1.84 ±0.03	1.38 ±0.04	2.15 ±0.13	1.55 ±0.11	1.65 ±0.05	1.66 ±0.04	2.05 ±0.14
	**3** [d]	12.85 ±0.06	14.21 ±0.12	12.17 ±0.10	12.77 ±0.11	11.79 ±0.07	10.78 ±0.16	13.80 ±0.26
	**4** [d]	1.89 ±0.12	1.84 ±0.06	1.73 ±0.00	1.64 ±0.15	1.73 ±0.05	1.57 ±0.05	1.91 ±0.08
	**5** [d]	6.14 ±0.03	6.59 ±0.11	5.77 ±0.18	5.69 ±0.17	5.83 ±0.23	5.19 ±0.23	5.94 ±0.19
	**6** [d]	7.16 ±0.03	6.95 ±0.13	6.89 ±0.22	7.07 ±0.17	7.04 ±0.03	6.39 ±0.11	9.50 ±0.17
	**7** [d]	2.09 ±0.07	1.46 ±0.12	1.32 ±0.08	0.96 ±0.15	1.19 ±0.14	1.02 ±0.18	1.32 ±0.19
	**8** [d]	0.85 ±0.07	0.75 ±0.14	0.99 ±0.17	0.67 ±0.14	0.70 ±0.15	0.73 ±0.17	0.94 ±0.06
	**9** [d]	3.66 ±0.03	2.76 ±0.06	4.09 ±0.10	2.97 ±0.28	3.13 ±0.07	3.02 ±0.16	4.05 ±0.11
	**10** [d]	0.91 ±0.07	0.73 ±0.11	1.33 ±0.05	0.86 ±0.06	0.93 ±0.07	0.89 ±0.09	1.26 ±0.10
	**11** [d]	0.24 ±0.01	0.21 ±0.10	0.26 ±0.04	0.16 ±0.04	0.19 ±0.04	0.18 ±0.10	0.25 ±0.07
	**12** [d]	0.69 ±0.05	0.65 ±0.09	1.22 ±0.14	0.80 ±0.09	0.81 ±0.08	0.92 ±0.07	1.07 ±0.12
80 ℃	**Moist.** [c]	63.24 ±0.21	28.63 ±0.46	14.23 ±0.30	6.06 ±0.31	4.10 ±0.15	4.00 ±0.23	2.90 ±0.15
	**1** [d]	2.10 ±0.07	1.67 ±0.05	1.89 ±0.05	1.79 ±0.05	1.57 ±0.10	1.63 ±0.03	1.88 ±0.07
	**2** [d]	1.84 ±0.03	1.39 ±0.06	1.82 ±0.01	2.09 ±0.04	1.80 ±0.01	1.80 ±0.06	2.05 ±0.18
	**3** [d]	12.85 ±0.06	12.34 ±0.06	12.73 ±0.12	13.47 ±0.12	11.88 ±0.10	13.26 ±0.05	11.91 ±0.09
	**4** [d]	1.89 ±0.12	1.73 ±0.11	1.79 ±0.04	2.06 ±0.04	2.17 ±0.06	2.02 ±0.03	1.79 ±0.10
	**5** [d]	6.14 ±0.03	5.93 ±0.06	5.92 ±0.09	6.36 ±0.10	5.85 ±0.09	6.09 ±0.07	5.65 ±0.15
	**6** [d]	7.16 ±0.03	6.54 ±0.00	7.13 ±0.06	7.72 ±0.11	7.64 ±0.13	7.25 ±0.11	7.17 ±0.14
	**7** [d]	2.09 ±0.07	1.75 ±0.03	1.15 ±0.02	1.23 ±0.03	1.43 ±0.04	1.30 ±0.06	1.16 ±0.02
	**8** [d]	0.85 ±0.07	0.80 ±0.06	0.83 ±0.03	0.87 ±0.08	0.94 ±0.11	0.85 ±0.03	0.90 ±0.03
	**9** [d]	3.66 ±0.03	3.08 ±0.01	3.30 ±0.06	3.73 ±0.03	3.95 ±0.07	3.56 ±0.12	4.01 ±0.04
	**10** [d]	0.91 ±0.07	0.86 ±0.02	1.06 ±0.03	1.28 ±0.01	1.19 ±0.03	1.15 ±0.06	1.31 ±0.11
	**11** [d]	0.24 ±0.01	0.21 ±0.01	0.27 ±0.01	0.30 ±0.01	0.23 ±0.02	0.27 ±0.02	0.29 ±0.04
	**12** [d]	0.69 ±0.05	0.77 ±0.05	0.96 ±0.03	1.16 ±0.03	1.13 ±0.00	1.01 ±0.02	1.12 ±0.08
90 ℃	**Moist.** [c]	63.24 ±0.21	19.96 ±0.16	7.33 ±0.19	3.03 ±0.12	1.89 ±0.33	1.79 ±0.36	1.33 ±0.14
	**1** [d]	2.10 ±0.07	2.00 ±0.03	1.73 ±0.03	1.89 ±0.03	2.08 ±0.21	1.62 ±0.18	1.75 ±0.04
	**2** [d]	1.84 ±0.03	2.22 ±0.01	1.59 ±0.04	1.86 ±0.03	1.88 ±0.21	1.66 ±0.16	1.72 ±0.11
	**3** [d]	12.85 ±0.06	14.47 ±0.02	13.69 ±0.08	14.09 ±0.05	13.36 ±0.07	12.76 ±0.04	11.48 ±0.12
	**4** [d]	1.89 ±0.12	1.91 ±0.05	2.01 ±0.05	2.15 ±0.04	2.00 ±0.21	1.81 ±0.13	1.70 ±0.07
	**5** [d]	6.14 ±0.03	7.34 ±0.02	6.12 ±0.04	6.69 ±0.12	6.33 ±0.02	5.93 ±0.29	5.83 ±0.22
	**6** [d]	7.16 ±0.03	7.98 ±0.06	7.48 ±0.05	8.20 ±0.11	8.28 ±0.25	6.92 ±0.06	6.87 ±0.21
	**7** [d]	2.09 ±0.07	1.99 ±0.04	1.04 ±0.03	1.23 ±0.05	1.22 ±0.04	1.74 ±0.21	1.33 ±0.12
	**8** [d]	0.85 ±0.07	1.19 ±0.05	0.79 ±0.02	1.00 ±0.03	0.92 ±0.10	0.98 ±0.06	0.94 ±0.08
	**9** [d]	3.66 ±0.03	5.12 ±0.10	3.36 ±0.08	4.06 ±0.05	3.91 ±0.14	3.88 ±0.06	4.09 ±0.24
	**10** [d]	0.91 ±0.07	1.49 ±0.07	1.06 ±0.04	1.22 ±0.05	1.30 ±0.08	1.10 ±0.08	1.22 ±0.13
	**11** [d]	0.24 ±0.01	0.41 ±0.05	0.26 ±0.01	0.31 ±0.00	0.29 ±0.02	0.27 ±0.13	0.28 ±0.04
	**12** [d]	0.69 ±0.05	1.53 ±0.09	0.91 ±0.03	1.20 ±0.01	1.07 ±0.08	0.94 ±0.12	1.16 ±0.03

[a] Results are presented as means ± sd (n = 3), Content is the value of dry weight; [b] Temperature; [c] Moisture (%); [d] units: mg/g Numbering of the compounds is the same as the Table S2-1.

Table S2-4. The data of rhizome of *B. chinensis* in **G1** from different sampling points at 100 ℃, 110 ℃ and 120 ℃. [a]

T. [b]	Analytes	Drying Time (min)						
		0	20	40	60	80	100	120
100 ℃	**Moist.** [c]	63.24 ±0.21	21.48 ±0.13	9.69 ±0.35	4.24 ±0.30	2.63 ±0.24	1.59 ±0.17	1.66 ±0.04
	**1** [d]	2.10 ±0.07	1.91 ±0.07	1.69 ±0.18	1.77 ±0.17	1.82 ±0.13	2.06 ±0.09	1.77 ±0.13
	**2** [d]	1.84 ±0.03	1.86 ±0.05	1.74 ±0.05	1.63 ±0.07	1.71 ±0.05	2.08 ±0.05	1.79 ±0.08
	**3** [d]	12.85 ±0.06	13.19 ±0.06	13.25 ±0.19	13.22 ±0.06	12.63 ±0.14	12.28 ±0.01	11.86 ±0.15
	**4** [d]	1.89 ±0.12	1.99 ±0.16	2.07 ±0.07	1.93 ±0.15	1.90 ±0.07	1.89 ±0.07	1.78 ±0.20
	**5** [d]	6.14 ±0.03	7.17 ±0.11	7.13 ±0.06	5.91 ±0.11	5.97 ±0.06	5.61 ±0.19	5.63 ±0.12
	**6** [d]	7.16 ±0.03	7.86 ±0.05	8.30 ±0.03	6.96 ±0.11	7.02 ±0.20	7.48 ±0.09	6.69 ±0.06
	**7** [d]	2.09 ±0.07	1.26 ±0.10	1.40 ±0.11	0.99 ±0.20	1.17 ±0.04	1.20 ±0.01	1.35 ±0.14
	**8** [d]	0.85 ±0.07	1.01 ±0.08	0.97 ±0.13	0.75 ±0.15	0.87 ±0.11	0.93 ±0.05	0.99 ±0.08
	**9** [d]	3.66 ±0.03	4.53 ±0.11	4.17 ±0.04	3.88 ±0.07	3.62 ±0.23	4.26 ±0.06	4.14 ±0.03
	**10** [d]	0.91 ±0.07	1.56 ±0.07	1.38 ±0.13	1.27 ±0.01	1.32 ±0.03	1.43 ±0.09	1.34 ±0.08
	**11** [d]	0.24 ±0.01	0.42 ±0.06	0.33 ±0.04	0.22 ±0.02	0.25 ±0.09	0.31 ±0.05	0.29 ±0.02
	**12** [d]	0.69 ±0.05	1.36 ±0.03	1.25 ±0.08	0.91 ±0.18	0.99 ±0.03	1.13 ±0.09	1.29 ±0.18
110 ℃	**Moist.** [c]	63.24 ±0.21	15.34 ±0.43	3.57 ±0.16	1.86 ±0.16	1.00 ±0.18	0.83 ±0.13	0.31 ±0.08
	**1** [d]	2.10 ±0.07	2.42 ±0.12	1.83 ±0.02	1.71 ±0.08	2.47 ±0.15	1.97 ±0.30	1.81 ±0.08
	**2** [d]	1.84 ±0.03	1.51 ±0.13	1.64 ±0.09	1.31 ±0.09	1.59 ±0.11	1.46 ±0.19	1.59 ±0.22
	**3** [d]	12.85 ±0.06	12.10 ±0.03	12.34 ±0.08	11.55 ±0.06	13.65 ±0.09	12.53 ±0.05	12.90 ±0.12
	**4** [d]	1.89 ±0.12	1.81 ±0.07	1.90 ±0.16	1.75 ±0.09	2.00 ±0.05	1.92 ±0.05	2.03 ±0.18
	**5** [d]	6.14 ±0.03	5.76 ±0.07	6.05 ±0.08	5.65 ±0.03	7.70 ±0.21	5.81 ±0.08	6.41 ±0.27
	**6** [d]	7.16 ±0.03	7.57 ±0.05	7.94 ±0.05	7.86 ±0.10	8.19 ±0.08	7.55 ±0.14	7.98 ±0.15
	**7** [d]	2.09 ±0.07	1.16 ±0.08	1.03 ±0.08	1.35 ±0.02	1.32 ±0.13	1.03 ±0.21	1.32 ±0.01
	**8** [d]	0.85 ±0.07	0.92 ±0.02	0.79 ±0.14	0.87 ±0.13	0.85 ±0.16	0.73 ±0.04	0.90 ±0.06
	**9** [d]	3.66 ±0.03	3.38 ±0.14	3.39 ±0.11	3.22 ±0.11	3.91 ±0.07	3.84 ±0.06	4.06 ±0.12
	**10** [d]	0.91 ±0.07	1.01 ±0.05	1.00 ±0.04	0.92 ±0.12	1.13 ±0.23	1.11 ±0.05	1.16 ±0.05
	**11** [d]	0.24 ±0.01	0.25 ±0.01	0.24 ±0.05	0.24 ±0.03	0.25 ±0.08	0.30 ±0.04	0.26 ±0.10
	**12** [d]	0.69 ±0.05	1.01 ±0.05	0.91 ±0.03	0.92 ±0.08	1.06 ±0.22	1.00 ±0.03	1.14 ±0.05
120 ℃	**Moist.** [c]	63.24 ±0.21	7.00 ±0.35	2.32 ±0.25	1.14 ±0.13	1.04 ±0.10	0.23 ±0.08	0.22 ±0.03
	**1** [d]	2.10 ±0.07	1.61 ±0.17	1.91 ±0.19	1.65 ±0.08	1.92 ±0.11	1.87 ±0.11	1.64 ±0.11
	**2** [d]	1.84 ±0.03	1.85 ±0.06	2.05 ±0.12	1.69 ±0.04	1.82 ±0.06	1.65 ±0.09	1.80 ±0.06
	**3** [d]	12.85 ±0.06	12.20 ±0.04	13.36 ±0.13	13.28 ±0.05	11.69 ±0.06	12.44 ±0.16	13.04 ±0.11
	**4** [d]	1.89 ±0.12	1.90 ±0.03	2.03 ±0.05	1.92 ±0.03	1.96 ±0.11	1.88 ±0.13	1.90 ±0.16
	**5** [d]	6.14 ±0.03	6.08 ±0.16	6.87 ±0.11	6.29 ±0.05	6.53 ±0.08	6.11 ±0.04	6.86 ±0.11
	**6** [d]	7.16 ±0.03	7.04 ±0.15	8.51 ±0.02	7.64 ±0.11	7.90 ±0.09	7.90 ±0.01	8.02 ±0.16
	**7** [d]	2.09 ±0.07	1.03 ±0.06	1.09 ±0.06	1.20 ±0.08	1.12 ±0.04	1.31 ±0.16	1.14 ±0.04
	**8** [d]	0.85 ±0.07	0.87 ±0.11	0.91 ±0.03	1.01 ±0.14	1.00 ±0.08	1.01 ±0.14	0.91 ±0.04
	**9** [d]	3.66 ±0.03	3.42 ±0.10	3.90 ±0.05	3.65 ±0.11	3.74 ±0.10	3.65 ±0.08	3.70 ±0.11
	**10** [d]	0.91 ±0.07	0.91 ±0.09	1.24 ±0.10	1.13 ±0.12	1.24 ±0.06	1.26 ±0.05	1.16 ±0.01
	**11** [d]	0.24 ±0.01	0.24 ±0.08	0.29 ±0.08	0.27 ±0.02	0.33 ±0.06	0.26 ±0.04	0.29 ±0.11
	**12** [d]	0.69 ±0.05	0.71 ±0.08	1.17 ±0.04	1.16 ±0.04	1.18 ±0.15	1.19 ±0.02	1.11 ±0.18

[a] Results are presented as means ± sd (n = 3), Content is the value of dry weight; [b] Temperature; [c] Moisture (%); [d] units: mg/g. Numbering of the compounds is the same as the Table S2-1.

Table S3-1. The data of rhizome of *B. chinensis* in **G2** from different sampling points at 40 ℃. [a]

T. [b]	Analytes	Drying Time (min)					
		0	120	240	360	480	600
40 ℃	**Moist.** [c]	63.99 ± 0.18	37.86 ± 0.17	28.19 ± 0.07	20.06 ± 0.11	11.08 ± 0.09	9.03 ± 0.12
	**1** [d]	1.47 ± 0.04	0.89 ± 0.00	0.78 ± 0.02	1.29 ± 0.02	1.27 ± 0.03	0.99 ± 0.00
	**2** [d]	0.94 ± 0.01	1.30 ± 0.03	1.04 ± 0.01	1.19 ± 0.08	1.20 ± 0.05	1.11 ± 0.07
	**3** [d]	11.87 ± 0.07	10.08 ± 0.05	11.38 ± 0.20	15.40 ± 0.29	13.77 ± 0.20	15.66 ± 0.27
	**4** [d]	1.66 ± 0.03	1.25 ± 0.03	1.36 ± 0.01	1.98 ± 0.05	1.81 ± 0.02	1.81 ± 0.01
	**5** [d]	6.21 ± 0.17	5.02 ± 0.03	5.38 ± 0.16	7.42 ± 0.17	7.20 ± 0.02	6.59 ± 0.11
	**6** [d]	5.85 ± 0.01	4.60 ± 0.01	4.82 ± 0.00	7.37 ± 0.10	6.84 ± 0.07	5.97 ± 0.01
	**7** [d]	2.47 ± 0.04	2.27 ± 0.04	1.32 ± 0.04	1.20 ± 0.04	1.31 ± 0.03	1.03 ± 0.02
	**8** [d]	0.63 ± 0.01	1.17 ± 0.00	0.60 ± 0.02	0.56 ± 0.02	0.56 ± 0.02	0.61 ± 0.03
	**9** [d]	1.73 ± 0.04	4.16 ± 0.00	2.10 ± 0.04	2.14 ± 0.08	2.05 ± 0.09	1.86 ± 0.00
	**10** [d]	0.50 ± 0.01	0.84 ± 0.00	0.76 ± 0.01	0.47 ± 0.02	0.41 ± 0.03	0.40 ± 0.01
	**11** [d]	0.13 ± 0.00	0.26 ± 0.00	0.34 ± 0.01	0.12 ± 0.01	0.14 ± 0.01	0.13 ± 0.01
	**12** [d]	0.55 ± 0.00	0.60 ± 0.00	0.93 ± 0.01	0.51 ± 0.02	1.15 ± 0.02	0.67 ± 0.00

[a] Results are presented as means ± sd (*n* = 3), Content is the value of dry weight; [b] Temperature; [c] Moisture (%); [d] units: mg/g. Numbering of the compounds is the same as the Table S2-1.

Table S3-2. The data of rhizome of *B. chinensis* in **G2** from different sampling points at 50 ℃ and 60 ℃. [a]

**T.** [b]	**Analytes**	**Drying Time (min)**								
		**0**	**60**	**120**	**180**	**240**	**300**	**360**	**420**	**480**
	**Moist.** [c]	63.99 ± 0.18	43.90 ± 0.44	22.96 ± 0.36	17.00 ± 0.51	16.68 ± 0.51	12.53 ± 0.32	10.67 ± 0.30	8.40 ± 0.15	8.26 ± 0.13
	**1** [d]	1.47 ± 0.04	1.02 ± 0.00	0.95 ± 0.02	1.12 ± 0.02	1.07 ± 0.03	0.90 ± 0.00	1.00 ± 0.02	0.98 ± 0.01	0.92 ± 0.03
	**2** [d]	0.94 ± 0.01	0.77 ± 0.02	1.35 ± 0.00	1.52 ± 0.04	1.43 ± 0.03	1.54 ± 0.00	1.25 ± 0.01	1.46 ± 0.01	1.49 ± 0.02
	**3** [d]	11.87 ± 0.07	11.33 ± 0.01	11.46 ± 0.02	13.08 ± 0.22	12.96 ± 0.06	12.24 ± 0.00	11.95 ± 0.19	12.11 ± 0.18	12.17 ± 0.14
	**4** [d]	1.66 ± 0.03	1.44 ± 0.03	1.56 ± 0.03	1.63 ± 0.03	1.58 ± 0.01	1.53 ± 0.00	1.51 ± 0.01	1.66 ± 0.03	1.67 ± 0.04
	**5** [d]	6.21 ± 0.17	5.65 ± 0.02	5.87 ± 0.04	6.44 ± 0.14	6.03 ± 0.04	6.22 ± 0.00	5.90 ± 0.06	6.20 ± 0.06	6.19 ± 0.06
**50 ℃**	**6** [d]	5.85 ± 0.01	5.66 ± 0.01	6.22 ± 0.01	6.40 ± 0.16	6.22 ± 0.13	6.73 ± 0.02	6.20 ± 0.06	6.65 ± 0.03	7.08 ± 0.03
	**7** [d]	2.47 ± 0.04	1.13 ± 0.00	0.88 ± 0.00	1.09 ± 0.00	0.86 ± 0.01	1.00 ± 0.00	0.92 ± 0.01	0.88 ± 0.03	1.06 ± 0.00
	**8** [d]	0.63 ± 0.01	0.63 ± 0.00	0.71 ± 0.01	0.78 ± 0.02	0.63 ± 0.01	0.61 ± 0.00	0.55 ± 0.02	0.63 ± 0.01	0.69 ± 0.01
	**9** [d]	1.73 ± 0.04	1.94 ± 0.03	2.47 ± 0.01	2.85 ± 0.04	2.39 ± 0.05	2.57 ± 0.03	2.20 ± 0.03	2.34 ± 0.06	2.76 ± 0.03
	**10** [d]	0.50 ± 0.01	0.46 ± 0.00	0.69 ± 0.00	0.78 ± 0.01	0.71 ± 0.02	0.68 ± 0.01	0.56 ± 0.02	0.67 ± 0.02	0.63 ± 0.01
	**11** [d]	0.13 ± 0.00	0.11 ± 0.00	0.17 ± 0.00	0.23 ± 0.01	0.19 ± 0.01	0.16 ± 0.01	0.12 ± 0.00	0.18 ± 0.01	0.17 ± 0.00
	**12** [d]	0.55 ± 0.00	0.58 ± 0.00	0.63 ± 0.01	0.76 ± 0.02	0.57 ± 0.02	0.71 ± 0.00	0.75 ± 0.03	0.55 ± 0.02	0.64 ± 0.02
	**Moist.** [c]	63.99 ± 0.18	46.96 ± 0.08	29.13 ± 0.21	14.69 ± 0.31	9.62 ± 0.17	7.44 ± 0.45	5.55 ± 0.17	5.63 ± 0.21	3.87 ± 0.39
	**1** [d]	1.47 ± 0.04	1.07 ± 0.05	1.03 ± 0.03	1.08 ± 0.00	1.12 ± 0.00	1.14 ± 0.01	1.08 ± 0.04	1.08 ± 0.03	1.03 ± 0.02
	**2** [d]	0.94 ± 0.01	1.26 ± 0.03	1.21 ± 0.01	1.43 ± 0.08	1.25 ± 0.05	1.17 ± 0.07	1.27 ± 0.08	1.27 ± 0.03	1.46 ± 0.06
	**3** [d]	11.87 ± 0.07	13.45 ± 0.22	13.09 ± 0.03	12.82 ± 0.20	14.59 ± 0.29	14.36 ± 0.29	13.62 ± 0.35	13.90 ± 0.27	12.38 ± 0.18
	**4** [d]	1.66 ± 0.03	1.64 ± 0.03	1.69 ± 0.02	1.81 ± 0.05	1.84 ± 0.01	1.81 ± 0.01	1.83 ± 0.05	1.72 ± 0.03	1.56 ± 0.01
	**5** [d]	6.21 ± 0.17	5.89 ± 0.07	5.90 ± 0.03	6.59 ± 0.07	6.99 ± 0.12	6.26 ± 0.09	6.33 ± 0.16	6.12 ± 0.10	5.60 ± 0.06
**60 ℃**	**6** [d]	5.85 ± 0.01	5.92 ± 0.08	5.89 ± 0.01	6.57 ± 0.13	7.32 ± 0.14	6.72 ± 0.11	7.01 ± 0.16	6.45 ± 0.17	5.87 ± 0.13
	**7** [d]	2.47 ± 0.04	1.45 ± 0.01	1.26 ± 0.03	1.40 ± 0.02	1.15 ± 0.01	1.19 ± 0.02	1.24 ± 0.00	1.20 ± 0.02	1.28 ± 0.03
	**8** d	0.63 ± 0.01	0.84 ± 0.01	0.77 ± 0.02	0.91 ± 0.01	0.70 ± 0.02	0.68 ± 0.00	0.73 ± 0.01	0.70 ± 0.01	0.77 ± 0.02
	**9** [d]	1.73 ± 0.04	2.63 ± 0.03	2.41 ± 0.04	2.91 ± 0.07	2.47 ± 0.06	2.36 ± 0.04	2.74 ± 0.05	2.59 ± 0.04	2.91 ± 0.07
	**10** [d]	0.50 ± 0.01	0.60 ± 0.01	0.61 ± 0.02	0.76 ± 0.02	0.64 ± 0.01	0.58 ± 0.02	0.69 ± 0.02	0.65 ± 0.02	0.79 ± 0.02
	**11** [d]	0.13 ± 0.00	0.17 ± 0.01	0.18 ± 0.01	0.23 ± 0.01	0.16 ± 0.01	0.17 ± 0.00	0.17 ± 0.01	0.17 ± 0.01	0.22 ± 0.01
	**12** [d]	0.55 ± 0.00	0.55 ± 0.02	0.55 ± 0.01	0.65 ± 0.01	0.73 ± 0.01	0.75 ± 0.01	0.59 ± 0.01	0.68 ± 0.02	0.73 ± 0.01

[a] Results are presented as means ± sd (n = 3), Content is the value of dry weight; [b] Temperature; [c] Moisture (%); [d] units: mg/g. Numbering of the compounds is the same as the Table S2-1.

Table S3-3. The data of rhizome of *B. chinensis* in **G2** from different sampling points at 70 ℃, 80 ℃ and 90 ℃. [a]

T. [b]	Analytes	Drying Time (min)						
		**0**	**30**	**60**	**90**	**120**	**150**	**180**
70 ℃	**Moist.** [c]	63.99 ± 0.18	45.59 ± 0.40	30.62 ± 0.43	15.76 ± 0.43	10.39 ± 0.12	8.39 ± 0.23	7.96 ± 0.25
	**1** [d]	1.47 ± 0.04	0.89 ± 0.03	0.98 ± 0.01	1.08 ± 0.03	1.09 ± 0.02	1.01 ± 0.01	0.97 ± 0.01
	**2** d	0.94 ± 0.01	1.13 ± 0.00	1.22 ± 0.03	1.90 ± 0.03	1.68 ± 0.04	1.28 ± 0.01	1.28 ± 0.03
	**3** d	11.87 ± 0.07	9.91 ± 0.05	9.79 ± 0.20	12.31 ± 0.29	13.03 ± 0.20	11.86 ± 0.27	10.49 ± 0.01
	**4** [d]	1.66 ± 0.03	1.15 ± 0.02	1.27 ± 0.01	1.66 ± 0.03	1.59 ± 0.04	1.59 ± 0.01	1.37 ± 0.03
	**5** [d]	6.21 ± 0.17	4.86 ± 0.09	4.74 ± 0.07	6.53 ± 0.13	6.81 ± 0.16	6.40 ± 0.13	5.26 ± 0.02
	**6** [d]	5.85 ± 0.01	3.93 ± 0.09	4.52 ± 0.12	6.95 ± 0.14	7.07 ± 0.13	6.69 ± 0.10	5.36 ± 0.01
	**7** [d]	2.47 ± 0.04	1.47 ± 0.04	1.72 ± 0.04	1.44 ± 0.04	1.26 ± 0.03	1.04 ± 0.02	1.18 ± 0.03
	**8** [d]	0.63 ± 0.01	1.07 ± 0.03	0.97 ± 0.02	0.99 ± 0.00	0.77 ± 0.01	0.73 ± 0.02	0.76 ± 0.01
	**9** [d]	1.73 ± 0.04	3.45 ± 0.06	3.56 ± 0.06	3.73 ± 0.07	3.07 ± 0.07	2.61 ± 0.06	2.61 ± 0.06
	**10** [d]	0.50 ± 0.01	0.43 ± 0.01	0.65 ± 0.02	0.99 ± 0.02	0.83 ± 0.02	0.64 ± 0.01	0.63 ± 0.03
	**11** [d]	0.13 ± 0.00	0.10 ± 0.00	0.17 ± 0.01	0.28 ± 0.00	0.19 ± 0.01	0.16 ± 0.00	0.14 ± 0.01
	**12** [d]	0.55 ± 0.00	0.52 ± 0.01	0.63 ± 0.01	0.98 ± 0.03	0.74 ± 0.04	0.62 ± 0.01	0.53 ± 0.01
80 ℃	**Moist.** c	63.99 ± 0.18	35.40 ± 0.32	15.16 ± 0.49	8.51 ± 0.31	6.21 ± 0.29	4.46 ± 0.37	4.49 ± 0.35
	**1**[d]	1.47 ± 0.04	1.04 ± 0.00	1.17 ± 0.03	1.13 ± 0.04	0.94 ± 0.01	1.17 ± 0.01	1.07 ± 0.03
	**2** [d]	0.94 ± 0.01	1.02 ± 0.01	1.73 ± 0.03	1.62 ± 0.03	1.42 ± 0.03	1.40 ± 0.02	1.48 ± 0.04
	**3** [d]	11.87 ± 0.07	13.17 ± 0.29	10.97 ± 0.22	11.48 ± 0.32	11.06 ± 0.08	11.57 ± 0.28	11.44 ± 0.06
	**4** [d]	1.66 ± 0.03	1.60 ± 0.03	1.61 ± 0.01	1.73 ± 0.05	1.57 ± 0.02	1.54 ± 0.01	1.51 ± 0.00
	**5** [d]	6.21 ± 0.17	5.28 ± 0.03	5.83 ± 0.16	6.28 ± 0.17	5.66 ± 0.02	5.79 ± 0.11	5.66 ± 0.03
	**6** [d]	5.85 ± 0.01	5.54 ± 0.05	6.76 ± 0.15	7.07 ± 0.14	6.58 ± 0.03	5.84 ± 0.22	5.75 ± 0.03
	**7** [d]	2.47 ± 0.04	1.38 ± 0.02	1.24 ± 0.01	1.15 ± 0.02	1.31 ± 0.02	1.23 ± 0.02	1.29 ± 0.01
	**8** [d]	0.63 ± 0.01	0.68 ± 0.03	0.87 ± 0.03	0.83 ± 0.03	0.81 ± 0.03	0.80 ± 0.02	0.88 ± 0.02
	**9** [d]	1.73 ± 0.04	2.06 ± 0.03	3.40 ± 0.09	3.18 ± 0.02	3.10 ± 0.07	3.07 ± 0.01	3.20 ± 0.06
	**10** [d]	0.50 ± 0.01	0.43 ± 0.01	0.87 ± 0.02	0.88 ± 0.01	0.82 ± 0.02	0.85 ± 0.01	0.87 ± 0.01
	**11** [d]	0.13 ± 0.00	0.12 ± 0.00	0.22 ± 0.01	0.20 ± 0.01	0.18 ± 0.01	0.20 ± 0.01	0.22 ± 0.01
	**12** [d]	0.55 ± 0.00	0.55 ± 0.01	0.84 ± 0.01	0.96 ± 0.04	0.88 ± 0.04	0.83 ± 0.01	0.87 ± 0.04
90 ℃	**Moist.** c	63.99 ± 0.18	28.32 ± 0.15	9.53 ± 0.23	5.38 ± 0.20	3.24 ± 0.23	2.48 ± 0.14	2.57 ± 0.41
	**1**[d]	1.47 ± 0.04	1.20 ± 0.00	1.10 ± 0.05	1.19 ± 0.04	1.10 ± 0.04	1.23 ± 0.01	1.07 ± 0.02
	**2** [d]	0.94 ± 0.01	1.51 ± 0.00	1.22 ± 0.03	1.76 ± 0.00	1.60 ± 0.05	1.48 ± 0.00	1.36 ± 0.00
	**3** [d]	11.87 ± 0.07	10.41 ± 0.01	11.56 ± 0.12	12.42 ± 0.27	11.59 ± 0.14	13.17 ± 0.01	10.54 ± 0.12
	**4** [d]	1.66 ± 0.03	1.72 ± 0.00	1.62 ± 0.01	1.78 ± 0.04	1.58 ± 0.03	1.75 ± 0.01	1.46 ± 0.02
	**5** [d]	6.21 ± 0.17	5.22 ± 0.00	5.66 ± 0.03	6.12 ± 0.15	5.53 ± 0.05	6.21 ± 0.01	5.39 ± 0.06
	**6** [d]	5.85 ± 0.01	6.09 ± 0.01	6.25 ± 0.00	6.88 ± 0.10	6.10 ± 0.07	6.34 ± 0.01	5.62 ± 0.03
	**7** [d]	2.47 ± 0.04	1.28 ± 0.00	1.22 ± 0.01	1.54 ± 0.01	1.14 ± 0.03	1.21 ± 0.00	1.40 ± 0.00
	**8** [d]	0.63 ± 0.01	0.98 ± 0.03	0.83 ± 0.02	1.13 ± 0.03	0.90 ± 0.04	0.89 ± 0.00	0.91 ± 0.04
	**9** [d]	1.73 ± 0.04	3.42 ± 0.00	2.78 ± 0.04	4.06 ± 0.08	3.37 ± 0.09	3.24 ± 0.00	3.27 ± 0.07
	**10** [d]	0.50 ± 0.01	0.98 ± 0.00	0.90 ± 0.02	1.18 ± 0.02	0.89 ± 0.01	0.83 ± 0.00	0.80 ± 0.02
	**11** [d]	0.13 ± 0.00	0.26 ± 0.00	0.28 ± 0.01	0.33 ± 0.01	0.23 ± 0.01	0.19 ± 0.00	0.23 ± 0.01
	**12** [d]	0.55 ± 0.00	0.85 ± 0.00	0.73 ± 0.01	0.92 ± 0.01	0.75 ± 0.03	0.72 ± 0.00	0.83 ± 0.01

[a] Results are presented as means ± sd (n = 3), Content is the value of dry weight; [b] Temperature; [c] Moisture (%); [d] units: mg/g. Numbering of the compounds is the same as the Table S2-1.

Table S3-4. The data of rhizome of *B. chinensis* in **G2** from different sampling points at 100 ℃, 110 ℃ and 120 ℃. [a]

T.	Analytes	Drying Time (min)						
		0	20	40	60	80	100	120
100 ℃	**Moist.** [c]	63.99 ± 0.18	26.83 ± 0.78	17.37 ± 0.28	6.78 ± 0.11	5.56 ± 0.25	3.47 ± 0.30	2.72 ± 0.20
	**1** [d]	1.47 ± 0.04	0.99 ± 0.00	0.97 ± 0.02	1.19 ± 0.01	1.65 ± 0.04	1.17 ± 0.03	1.05 ± 0.04
	**2** [d]	0.94 ± 0.01	1.71 ± 0.00	1.26 ± 0.02	1.64 ± 0.01	1.63 ± 0.02	1.44 ± 0.03	1.36 ± 0.02
	**3** [d]	11.87 ± 0.07	10.17 ± 0.00	11.32 ± 0.08	12.51 ± 0.22	14.17 ± 0.03	11.91 ± 0.05	11.14 ± 0.21
	**4** [d]	1.66 ± 0.03	1.33 ± 0.01	1.54 ± 0.01	1.69 ± 0.02	1.88 ± 0.03	1.59 ± 0.04	1.41 ± 0.03
	**5** [d]	6.21 ± 0.17	5.26 ± 0.00	5.47 ± 0.07	6.41 ± 0.07	7.07 ± 0.19	5.50 ± 0.12	4.86 ± 0.11
	**6** [d]	5.85 ± 0.01	5.32 ± 0.00	5.96 ± 0.11	6.93 ± 0.07	7.54 ± 0.18	6.76 ± 0.12	5.22 ± 0.11
	**7** [d]	2.47 ± 0.04	1.47 ± 0.00	0.93 ± 0.02	1.02 ± 0.00	1.35 ± 0.02	1.09 ± 0.01	1.07 ± 0.03
	**8** [d]	0.63 ± 0.01	1.00 ± 0.00	0.67 ± 0.02	0.75 ± 0.02	0.95 ± 0.02	0.84 ± 0.03	0.94 ± 0.00
	**9** [d]	1.73 ± 0.04	3.88 ± 0.00	2.97 ± 0.05	2.94 ± 0.06	3.47 ± 0.08	2.94 ± 0.06	2.92 ± 0.05
	**10** [d]	0.50 ± 0.01	1.14 ± 0.00	0.80 ± 0.01	0.81 ± 0.02	0.98 ± 0.03	0.80 ± 0.01	0.80 ± 0.02
	**11** [d]	0.13 ± 0.00	0.25 ± 0.00	0.16 ± 0.01	0.19 ± 0.01	0.21 ± 0.01	0.22 ± 0.01	0.23 ± 0.01
	**12** [d]	0.55 ± 0.00	0.89 ± 0.00	0.68 ± 0.03	0.69 ± 0.01	0.78 ± 0.03	0.64 ± 0.02	0.63 ± 0.01
110 ℃	**Moist.** c	63.99 ± 0.18	14.37 ± 0.24	4.41 ± 0.38	1.66 ± 0.26	1.19 ± 0.15	0.77 ± 0.03	0.45 ± 0.26
	**1** [d]	1.47 ± 0.04	1.36 ± 0.01	1.23 ± 0.01	1.02 ± 0.02	1.08 ± 0.00	1.09 ± 0.03	1.16 ± 0.04
	**2** [d]	0.94 ± 0.01	1.73 ± 0.00	1.54 ± 0.02	1.56 ± 0.04	1.60 ± 0.02	1.74 ± 0.07	1.52 ± 0.04
	**3** [d]	11.87 ± 0.07	13.76 ± 0.07	11.71 ± 0.06	11.55 ± 0.14	10.82 ± 0.16	11.41 ± 0.18	11.47 ± 0.22
	**4** [d]	1.66 ± 0.03	1.95 ± 0.01	1.62 ± 0.03	1.48 ± 0.03	1.55 ± 0.01	1.47 ± 0.02	1.58 ± 0.02
	**5** [d]	6.21 ± 0.17	6.98 ± 0.14	5.86 ± 0.10	6.14 ± 0.16	5.51 ± 0.02	5.87 ± 0.02	6.17 ± 0.22
	**6** [d]	5.85 ± 0.01	7.48 ± 0.18	6.17 ± 0.16	6.44 ± 0.11	6.58 ± 0.08	5.77 ± 0.06	6.84 ± 0.15
	**7** [d]	2.47 ± 0.04	0.97 ± 0.01	0.92 ± 0.01	0.98 ± 0.01	1.03 ± 0.03	0.88 ± 0.02	0.95 ± 0.02
	**8** [d]	0.63 ± 0.01	0.78 ± 0.01	0.68 ± 0.02	0.85 ± 0.03	0.78 ± 0.00	0.67 ± 0.01	0.73 ± 0.02
	**9** [d]	1.73 ± 0.04	3.11 ± 0.01	2.73 ± 0.02	3.40 ± 0.06	2.86 ± 0.01	2.46 ± 0.04	2.77 ± 0.04
	**10** [d]	0.50 ± 0.01	0.87 ± 0.01	0.91 ± 0.02	1.02 ± 0.04	0.80 ± 0.00	0.75 ± 0.03	0.83 ± 0.02
	**11** [d]	0.13 ± 0.00	0.25 ± 0.01	0.20 ± 0.01	0.25 ± 0.01	0.22 ± 0.01	0.20 ± 0.01	0.19 ± 0.01
	**12** [d]	0.55 ± 0.00	0.87 ± 0.00	0.74 ± 0.03	0.80 ± 0.01	0.70 ± 0.00	0.63 ± 0.01	0.63 ± 0.02
120 ℃	**Moist.** c	63.99 ± 0.18	5.53 ± 0.31	1.73 ± 0.08	0.29 ± 0.08	0.31 ± 0.13	0.21 ± 0.04	0.19 ± 0.02
	**1**[d]	1.47 ± 0.04	1.18 ± 0.03	1.35 ± 0.05	1.24 ± 0.02	1.13 ± 0.04	1.12 ± 0.04	1.13 ± 0.05
	**2** [d]	0.94 ± 0.01	1.02 ± 0.07	1.81 ± 0.03	3.84 ± 0.10	2.18 ± 0.02	2.65 ± 0.09	1.61 ± 0.06
	**3** [d]	11.87 ± 0.07	11.34 ± 0.29	12.49 ± 0.18	12.21 ± 0.18	11.83 ± 0.23	10.70 ± 0.19	11.27 ± 0.16
	**4** [d]	1.66 ± 0.03	1.55 ± 0.04	1.64 ± 0.01	1.75 ± 0.01	1.65 ± 0.05	1.56 ± 0.04	1.48 ± 0.04
	**5** [d]	6.21 ± 0.17	5.52 ± 0.13	6.03 ± 0.09	5.63 ± 0.07	5.52 ± 0.16	5.74 ± 0.07	5.13 ± 0.07
	**6** [d]	5.85 ± 0.01	6.89 ± 0.11	6.86 ± 0.09	6.75 ± 0.07	6.25 ± 0.12	6.53 ± 0.09	5.77 ± 0.03
	**7** [d]	2.47 ± 0.04	1.06 ± 0.03	0.89 ± 0.03	0.99 ± 0.01	1.20 ± 0.03	1.07 ± 0.02	1.04 ± 0.02
	**8** [d]	0.63 ± 0.01	0.78 ± 0.02	0.74 ± 0.02	0.78 ± 0.01	0.83 ± 0.04	0.87 ± 0.02	0.77 ± 0.02
	**9** [d]	1.73 ± 0.04	1.86 ± 0.04	2.87 ± 0.05	2.83 ± 0.06	3.17 ± 0.05	3.42 ± 0.05	2.77 ± 0.07
	**10** [d]	0.50 ± 0.01	0.77 ± 0.03	0.74 ± 0.01	0.71 ± 0.02	0.73 ± 0.01	0.90 ± 0.03	0.66 ± 0.01
	**11** [d]	0.13 ± 0.00	0.19 ± 0.01	0.22 ± 0.01	0.22 ± 0.01	0.22 ± 0.01	0.23 ± 0.00	0.19 ± 0.01
	**12** [d]	0.55 ± 0.00	0.56 ± 0.02	0.60 ± 0.01	0.72 ± 0.02	0.83 ± 0.01	0.97 ± 0.02	0.74 ± 0.03

[a] Results are presented as means ± sd (n = 3), Content is the value of dry weight; [b] Temperature; [c] Moisture (%); [d] units: mg/g. Numbering of the compounds is the same as the Table S2-1.

Table S4-1. The data of rhizome of *B. chinensis* in **G3** from different sampling points at 40 ℃. [a]

T. [b]	Analytes	Drying Time (min)					
		0	120	240	360	480	600
40 °C	**Moist.** [c]	61.59 ± 0.63	39.98 ± 0.16	26.88 ± 0.14	18.71 ± 0.17	10.24 ± 0.10	8.57 ± 0.13
	**1** [d]	1.08 ± 0.06	1.07 ± 0.02	1.02 ± 0.07	1.06 ± 0.04	1.14 ± 0.05	1.08 ± 0.01
	**2** [d]	1.40 ± 0.04	0.89 ± 0.04	1.39 ± 0.00	1.48 ± 0.00	1.37 ± 0.00	1.53 ± 0.00
	**3** [d]	9.65 ± 0.03	9.66 ± 0.10	10.95 ± 0.04	15.34 ± 0.00	14.51 ± 0.00	13.64 ± 0.01
	**4** [d]	1.48 ± 0.07	1.24 ± 0.19	1.50 ± 0.06	1.96 ± 0.00	1.75 ± 0.00	1.75 ± 0.00
	**5** [d]	5.82 ± 0.18	4.33 ± 0.00	5.98 ± 0.02	6.75 ± 0.00	6.41 ± 0.06	6.28 ± 0.03
	**6** [d]	5.70 ± 0.19	3.62 ± 0.00	5.64 ± 0.05	6.15 ± 0.05	5.70 ± 0.00	5.81 ± 0.02
	**7** [d]	2.41 ± 0.04	2.20 ± 0.00	1.51 ± 0.18	1.40 ± 0.06	1.30 ± 0.00	1.38 ± 0.11
	**8** [d]	0.76 ± 0.02	1.17 ± 0.03	0.68 ± 0.00	0.72 ± 0.03	0.65 ± 0.00	0.69 ± 0.00
	**9** [d]	2.57 ± 0.09	3.66 ± 0.07	2.82 ± 0.00	2.69 ± 0.29	2.61 ± 0.00	2.85 ± 0.07
	**10** [d]	0.54 ± 0.03	0.40 ± 0.00	0.56 ± 0.15	0.44 ± 0.01	0.43 ± 0.00	0.50 ± 0.02
	**11** [d]	0.17 ± 0.01	0.19 ± 0.00	0.14 ± 0.00	0.14 ± 0.00	0.12 ± 0.00	0.14 ± 0.00
	**12** [d]	0.55 ± 0.04	0.38 ± 0.03	0.51 ± 0.02	0.81 ± 0.04	0.46 ± 0.02	0.49 ± 0.04

[a] Results are presented as means ± sd (n = 3), Content is the value of dry weight; [b] Temperature; [c] Moisture (%); [d] units: mg/g. Numbering of the compounds is the same as the Table S2-1.

Table S4-2. The data of rhizome of *B. chinensis* in **G3** from different sampling points at 50 ℃ and 60 ℃. [a]

**T.** [b]	**Analytes**	**Drying Time (min)**								
		**0**	**60**	**120**	**180**	**240**	**300**	**360**	**420**	**480**
**50 ℃**	**Moist.** [c]	61.59 ±0.63	47.36 ±0.36	36.57 ±0.24	23.15 ±0.38	18.52 ±0.32	12.40 ±0.27	11.43 ±0.23	7.57 ±0.09	7.28 ±0.29
	**1** [d]	1.08 ±0.06	1.12 ±0.02	1.17 ±0.07	1.12 ±0.04	1.09 ±0.05	1.06 ±0.01	1.08 ±0.04	1.07 ±0.05	0.93 ±0.00
	**2** [d]	1.40 ±0.04	1.61 ±0.02	1.60 ±0.01	2.93 ±0.10	2.26 ±0.06	2.37 ±0.02	2.08 ±0.00	1.87 ±0.00	1.71 ±0.01
	**3** [d]	9.65 ±0.13	12.10 ±0.00	12.13 ±0.04	12.51 ±0.04	12.89 ±0.16	11.71 ±0.00	11.77 ±0.00	11.46 ±0.00	11.42 ±0.00
	**4** [d]	1.48 ±0.07	1.65 ±0.02	1.69 ±0.00	1.66 ±0.06	1.93 ±0.07	1.71 ±0.00	1.80 ±0.00	1.68 ±0.00	1.63 ±0.00
	**5** [d]	5.82 ±0.18	6.20 ±0.01	6.13 ±0.00	6.53 ±0.20	6.94 ±0.09	6.63 ±0.00	7.13 ±0.00	6.43 ±0.00	6.08 ±0.00
	**6** [d]	5.70 ±0.19	5.53 ±0.00	5.59 ±0.00	6.02 ±0.19	6.49 ±0.10	6.55 ±0.00	6.93 ±0.01	6.40 ±0.00	5.84 ±0.00
	**7** [d]	2.41 ±0.04	1.40 ±0.00	1.69 ±0.00	2.05 ±0.01	1.48 ±0.07	1.73 ±0.00	1.75 ±0.00	1.23 ±0.00	1.18 ±0.00
	**8** [d]	0.76 ±0.02	0.85 ±0.00	0.90 ±0.00	1.54 ±0.01	1.07 ±0.05	1.32 ±0.26	1.06 ±0.00	0.76 ±0.00	0.75 ±0.00
	**9** [d]	2.57 ±0.09	3.00 ±0.00	3.42 ±0.00	6.19 ±0.12	4.22 ±0.11	4.61 ±0.04	4.49 ±0.00	3.20 ±0.01	3.18 ±0.00
	**10** [d]	0.54 ±0.03	0.61 ±0.01	0.62 ±0.00	1.44 ±0.08	0.98 ±0.05	0.90 ±0.00	0.80 ±0.00	0.62 ±0.01	0.57 ±0.00
	**11** [d]	0.17 ±0.01	0.18 ±0.00	0.17 ±0.00	0.39 ±0.05	0.27 ±0.02	0.22 ±0.00	0.20 ±0.00	0.16 ±0.00	0.16 ±0.00
	**12** [d]	0.55 ±0.04	0.56 ±0.00	0.58 ±0.00	1.75 ±0.04	0.89 ±0.03	0.86 ±0.00	0.85 ±0.00	0.63 ±0.00	0.59 ±0.01
**60 ℃**	**Moist.** c	61.59 ±0.63	26.30 ±0.16	16.68 ±0.34	10.61 ±0.35	7.01 ±0.16	6.40 ±0.31	5.28 ±0.17	4.32 ±0.22	4.29 ±0.19
	**1** [d]	1.08 ±0.06	0.90 ±0.10	0.92 ±0.01	0.99 ±0.01	1.06 ±0.00	0.93 ±0.00	0.99 ±0.01	0.91 ±0.00	1.19 ±0.24
	**2** [d]	1.40 ±0.04	1.87 ±0.04	2.01 ±0.00	1.69 ±0.00	1.96 ±0.00	1.90 ±0.00	1.73 ±0.13	1.75 ±0.00	2.00 ±0.11
	**3** [d]	9.65 ±0.03	9.72 ±0.10	9.86 ±0.04	11.30 ±0.00	11.20 ±0.00	10.80 ±0.01	10.45 ±0.04	10.30 ±0.02	10.15 ±0.06
	**4** [d]	1.48 ±0.07	1.40 ±0.19	1.53 ±0.06	1.72 ±0.00	1.70 ±0.00	1.51 ±0.00	1.53 ±0.05	1.45 ±0.01	1.55 ±0.05
	**5** [d]	5.82 ±0.18	5.76 ±0.08	5.84 ±0.18	7.01 ±0.01	6.08 ±0.00	6.26 ±0.03	6.30 ±0.22	5.73 ±0.00	6.38 ±0.04
	**6** [d]	5.70 ±0.19	5.63 ±0.72	5.73 ±0.12	6.67 ±0.00	6.04 ±0.00	6.04 ±0.00	6.15 ±0.13	5.80 ±0.00	6.23 ±0.04
	**7** [d]	2.41 ±0.04	1.18 ±0.22	1.39 ±0.17	1.10 ±0.00	1.43 ±0.00	1.12 ±0.00	1.11 ±0.06	1.13 ±0.00	1.41 ±0.11
	**8** [d]	0.76 ±0.02	0.84 ±0.26	0.91 ±0.05	0.76 ±0.02	0.83 ±0.00	0.83 ±0.00	0.77 ±0.11	0.78 ±0.02	1.09 ±0.21
	**9** [d]	2.57 ±0.09	3.60 ±0.17	3.93 ±0.12	3.46 ±0.00	3.73 ±0.00	3.64 ±0.00	3.47 ±0.04	3.33 ±0.00	4.41 ±0.39
	**10** [d]	0.54 ±0.03	0.81 ±0.28	0.78 ±0.02	0.67 ±0.00	0.71 ±0.00	0.82 ±0.00	0.72 ±0.10	0.69 ±0.00	0.75 ±0.02
	**11** [d]	0.17 ±0.01	0.21 ±0.03	0.23 ±0.00	0.17 ±0.00	0.18 ±0.00	0.22 ±0.00	0.20 ±0.03	0.21 ±0.00	0.20 ±0.01
	**12** [d]	0.55 ±0.02	0.51 ±0.01	0.77 ±0.02	0.69 ±0.01	0.74 ±0.00	0.75 ±0.01	0.73 ±0.06	0.70 ±0.00	0.77 ±0.05

[a] Results are presented as means ± sd (n = 3), Content is the value of dry weight; [b] Temperature; [c] Moisture (%); [d] units: mg/g. Numbering of the compounds is the same as the Table S2-1.

Table S4-3. The data of rhizome of *B. chinensis* in **G3** from different sampling points at 70 ℃, 80 ℃ and 90 ℃. [a]

T. [b]	Analytes	Drying Time (min)						
		0	30	60	90	120	150	180
70 ℃	Moist. [c]	61.59 ± 0.63	43.32 ± 0.55	35.61 ± 0.41	18.87 ± 0.28	9.51 ± 0.43	8.34 ± 0.37	6.34 ± 0.22
	1 [d]	1.08 ± 0.06	0.97 ± 0.00	0.92 ± 0.01	1.38 ± 0.00	1.38 ± 0.01	1.24 ± 0.36	0.78 ± 0.02
	2 [d]	1.40 ± 0.04	1.11 ± 0.00	1.24 ± 0.00	2.05 ± 0.00	1.68 ± 0.18	1.89 ± 0.00	1.62 ± 0.02
	3 [d]	9.65 ± 0.03	9.75 ± 0.00	10.71 ± 0.00	11.93 ± 0.00	10.85 ± 0.10	11.43 ± 0.87	9.56 ± 0.11
	4 [d]	1.48 ± 0.07	1.18 ± 0.00	1.35 ± 0.00	1.89 ± 0.00	1.44 ± 0.14	1.55 ± 0.10	1.34 ± 0.01
	5 [d]	5.82 ± 0.18	4.62 ± 0.00	5.39 ± 0.02	7.38 ± 0.00	6.25 ± 0.06	6.19 ± 0.03	5.35 ± 0.04
	6 [d]	5.70 ± 0.19	4.30 ± 0.00	4.80 ± 0.01	7.25 ± 0.00	5.58 ± 0.05	5.73 ± 0.31	5.11 ± 0.03
	7 [d]	2.41 ± 0.04	2.03 ± 0.00	2.12 ± 0.00	1.36 ± 0.00	1.55 ± 0.19	1.52 ± 0.03	1.42 ± 0.03
	8 [d]	0.76 ± 0.02	1.04 ± 0.00	0.97 ± 0.00	0.98 ± 0.00	0.88 ± 0.09	0.86 ± 0.01	0.86 ± 0.07
	9 [d]	2.57 ± 0.09	3.45 ± 0.00	3.50 ± 0.01	4.16 ± 0.00	3.86 ± 0.05	3.78 ± 0.04	3.85 ± 0.23
	10 [d]	0.54 ± 0.03	0.49 ± 0.00	0.41 ± 0.00	0.67 ± 0.00	0.59 ± 0.05	0.58 ± 0.00	0.56 ± 0.03
	11 [d]	0.17 ± 0.01	0.17 ± 0.00	0.14 ± 0.00	0.20 ± 0.00	0.20 ± 0.00	0.19 ± 0.00	0.19 ± 0.01
	12 [d]	0.55 ± 0.04	0.52 ± 0.00	0.51 ± 0.01	0.66 ± 0.00	0.71 ± 0.00	0.61 ± 0.03	0.68 ± 0.03
80 ℃	Moist. c	61.59 ± 0.63	28.54 ± 0.34	13.32 ± 0.36	8.16 ± 0.31	5.15 ± 0.15	4.00 ± 0.17	3.14 ± 0.40
	1 [d]	1.08 ± 0.06	1.19 ± 0.00	0.98 ± 0.06	1.00 ± 0.05	0.88 ± 0.00	0.96 ± 0.02	0.89 ± 0.01
	2 [d]	1.40 ± 0.04	1.16 ± 0.00	1.81 ± 0.23	1.61 ± 0.06	1.71 ± 0.00	1.65 ± 0.11	1.70 ± 0.13
	3 [d]	9.65 ± 0.33	11.37 ± 0.00	10.05 ± 0.08	11.41 ± 0.50	10.83 ± 0.00	11.20 ± 0.08	10.79 ± 0.20
	4 [d]	1.48 ± 0.07	1.62 ± 0.00	1.49 ± 0.03	1.58 ± 0.04	1.64 ± 0.00	1.54 ± 0.03	1.48 ± 0.02
	5 [d]	5.82 ± 0.18	5.88 ± 0.00	6.16 ± 0.00	6.02 ± 0.23	5.85 ± 0.00	6.18 ± 0.21	6.03 ± 0.39
	6 [d]	5.70 ± 0.19	5.71 ± 0.00	5.63 ± 0.05	5.65 ± 0.05	5.97 ± 0.00	5.73 ± 0.02	6.05 ± 0.06
	7 [d]	2.41 ± 0.04	0.94 ± 0.00	1.26 ± 0.18	1.14 ± 0.06	1.17 ± 0.00	1.26 ± 0.11	1.30 ± 0.19
	8 [d]	0.76 ± 0.02	0.74 ± 0.00	0.94 ± 0.21	0.78 ± 0.03	0.81 ± 0.00	0.87 ± 0.10	0.89 ± 0.06
	9 [d]	2.57 ± 0.09	2.05 ± 0.00	3.74 ± 0.07	3.26 ± 0.01	3.54 ± 0.00	3.73 ± 0.35	3.63 ± 0.06
	10 [d]	0.54 ± 0.03	0.34 ± 0.00	0.64 ± 0.15	0.55 ± 0.01	0.61 ± 0.00	0.60 ± 0.02	0.65 ± 0.02
	11 [d]	0.17 ± 0.01	0.09 ± 0.00	0.23 ± 0.00	0.17 ± 0.00	0.19 ± 0.00	0.19 ± 0.00	0.23 ± 0.02
	12 [d]	0.55 ± 0.04	0.43 ± 0.03	0.86 ± 0.00	0.62 ± 0.00	0.66 ± 0.00	0.67 ± 0.02	0.72 ± 0.01
90 ℃	Moist. c	61.59 ± 0.63	26.53 ± 0.38	12.37 ± 0.26	5.44 ± 0.30	3.38 ± 0.29	2.62 ± 0.25	2.44 ± 0.38
	1 [d]	1.08 ± 0.06	1.02 ± 0.14	1.38 ± 0.00	1.05 ± 0.01	0.94 ± 0.00	1.46 ± 0.04	1.02 ± 0.01
	2 [d]	1.40 ± 0.04	1.33 ± 0.12	1.38 ± 0.01	1.82 ± 0.11	1.53 ± 0.02	1.70 ± 0.01	1.65 ± 0.10
	3 [d]	9.65 ± 0.33	10.66 ± 0.05	11.28 ± 0.00	11.24 ± 0.03	11.91 ± 0.02	12.24 ± 0.09	11.95 ± 0.05
	4 [d]	1.48 ± 0.07	1.50 ± 0.05	1.60 ± 0.00	1.79 ± 0.06	1.73 ± 0.01	1.78 ± 0.03	1.72 ± 0.05
	5 [d]	5.82 ± 0.18	6.03 ± 0.15	6.88 ± 0.02	7.18 ± 0.02	6.69 ± 0.00	6.83 ± 0.13	6.71 ± 0.02
	6 [d]	5.70 ± 0.19	5.47 ± 0.23	6.53 ± 0.00	6.86 ± 0.14	6.63 ± 0.00	6.90 ± 0.10	6.43 ± 0.09
	7 [d]	2.41 ± 0.04	1.09 ± 0.05	0.94 ± 0.00	1.07 ± 0.08	1.11 ± 0.00	1.23 ± 0.04	1.34 ± 0.01
	8 [d]	0.76 ± 0.02	0.64 ± 0.03	0.68 ± 0.00	0.81 ± 0.03	0.88 ± 0.00	0.87 ± 0.00	0.94 ± 0.07
	9 [d]	2.57 ± 0.09	2.58 ± 0.07	2.93 ± 0.00	3.63 ± 0.29	3.47 ± 0.00	3.52 ± 0.07	3.77 ± 0.19
	10 [d]	0.54 ± 0.03	0.44 ± 0.01	0.52 ± 0.00	0.69 ± 0.08	0.71 ± 0.00	0.69 ± 0.01	0.69 ± 0.04
	11 [d]	0.17 ± 0.01	0.14 ± 0.01	0.17 ± 0.00	0.20 ± 0.03	0.15 ± 0.00	0.20 ± 0.01	0.20 ± 0.00
	12 [d]	0.55 ± 0.04	0.55 ± 0.03	0.60 ± 0.02	0.59 ± 0.04	0.64 ± 0.02	0.60 ± 0.04	0.76 ± 0.04

[a] Results are presented as means ± sd (n = 3), Content is the value of dry weight; [b] Temperature; [c] Moisture (%); [d] units: mg/g. Numbering of the compounds is the same as the Table S2-1.

Table S4-4. The data of rhizome of *B. chinensis* in **G3** from different sampling points at 100 ℃, 110 ℃ and 120 ℃. [a]

T. [b]	Analytes	Drying Time (min)						
		0	20	40	60	80	100	120
100 ℃	Moist. [c]	61.59 ±0.63	23.79 ±0.31	10.39 ±0.23	5.62 ±0.15	4.28 ±1.02	2.94 ±0.17	2.48 ±0.18
	1 [d]	1.08 ±0.06	0.98 ±0.04	1.01 ±0.01	0.98 ±0.03	1.11 ±0.06	0.89 ±0.00	0.88 ±0.00
	2 [d]	1.40 ±0.04	1.39 ±0.04	1.72 ±0.04	1.56 ±0.12	2.08 ±0.03	1.67 ±0.00	1.81 ±0.00
	3 [d]	9.65 ±0.03	11.28 ±0.30	10.57 ±0.01	10.64 ±0.08	11.14 ±0.34	10.09 ±0.02	10.68 ±0.00
	4 [d]	1.48 ±0.07	1.47 ±0.03	1.62 ±0.00	1.57 ±0.05	1.55 ±0.08	1.52 ±0.00	1.55 ±0.00
	5 [d]	5.82 ±0.18	5.24 ±0.04	6.54 ±0.01	6.50 ±0.04	6.19 ±0.18	5.81 ±0.00	5.86 ±0.00
	6 [d]	5.70 ±0.19	5.39 ±0.05	6.39 ±0.04	5.96 ±0.14	5.85 ±0.12	5.64 ±0.00	5.77 ±0.00
	7 [d]	2.41 ±0.04	0.89 ±0.02	1.24 ±0.00	1.32 ±0.03	1.36 ±0.09	1.19 ±0.00	1.25 ±0.00
	8 [d]	0.76 ±0.02	0.63 ±0.03	0.90 ±0.02	0.90 ±0.05	1.06 ±0.09	0.87 ±0.00	0.87 ±0.00
	9 [d]	2.57 ±0.09	2.64 ±0.09	3.65 ±0.00	3.94 ±0.25	4.14 ±0.12	3.78 ±0.00	3.59 ±0.00
	10 [d]	0.54 ±0.03	0.47 ±0.02	0.67 ±0.00	0.72 ±0.02	0.78 ±0.03	0.72 ±0.01	0.65 ±0.00
	11 [d]	0.17 ±0.01	0.15 ±0.00	0.22 ±0.00	0.21 ±0.00	0.23 ±0.03	0.20 ±0.00	0.23 ±0.00
	12 [d]	0.55 ±0.04	0.56 ±0.03	0.82 ±0.02	0.87 ±0.00	0.77 ±0.05	0.83 ±0.04	0.82 ±0.00
110 ℃	Moist. c	61.59 ±0.63	12.46 ±0.30	4.00 ±0.25	1.63 ±0.22	0.97 ±0.04	0.87 ±0.09	0.71 ±0.16
	1 [d]	1.08 ±0.06	1.13 ±0.00	0.96 ±0.03	0.91 ±0.00	1.04 ±0.00	1.22 ±0.05	1.01 ±0.00
	2 [d]	1.40 ±0.04	1.80 ±0.00	1.82 ±0.08	2.15 ±0.00	1.79 ±0.00	1.87 ±0.20	1.85 ±0.01
	3 [d]	9.65 ±0.03	12.40 ±0.00	10.52 ±0.19	11.04 ±0.00	10.53 ±0.00	11.40 ±0.10	10.69 ±0.00
	4 [d]	1.48 ±0.07	1.97 ±0.00	1.59 ±0.04	1.67 ±0.00	1.59 ±0.00	1.71 ±0.09	1.61 ±0.01
	5 [d]	5.82 ±0.18	7.08 ±0.00	6.80 ±0.15	7.28 ±0.00	6.30 ±0.00	6.95 ±0.08	6.66 ±0.00
	6 [d]	5.70 ±0.19	7.82 ±0.00	6.89 ±0.23	7.32 ±0.00	6.52 ±0.00	7.35 ±0.14	6.77 ±0.00
	7 [d]	2.41 ±0.04	1.19 ±0.00	1.11 ±0.07	1.09 ±0.00	0.93 ±0.00	1.13 ±0.11	1.07 ±0.00
	8 [d]	0.76 ±0.02	0.88 ±0.00	0.87 ±0.08	0.83 ±0.00	0.72 ±0.00	0.84 ±0.13	0.88 ±0.00
	9 [d]	2.57 ±0.09	3.75 ±0.00	3.71 ±0.03	3.98 ±0.00	3.33 ±0.00	3.83 ±0.03	3.73 ±0.00
	10 [d]	0.54 ±0.03	0.86 ±0.00	0.79 ±0.10	0.77 ±0.00	0.66 ±0.00	0.85 ±0.11	0.81 ±0.00
	11 [d]	0.17 ±0.01	0.22 ±0.00	0.21 ±0.01	0.21 ±0.00	0.23 ±0.00	0.24 ±0.01	0.30 ±0.00
	12 [d]	0.55 ±0.04	1.01 ±0.00	0.93 ±0.01	0.83 ±0.00	0.83 ±0.00	0.89 ±0.02	0.77 ±0.00
120 ℃	Moist. c	61.59 ±0.63	5.99 ±0.19	1.37 ±0.38	0.35 ±0.13	0.37 ±0.17	0.22 ±0.03	0.27 ±0.08
	1 [d]	1.08 ±0.06	0.99 ±0.04	1.06 ±0.00	1.08 ±0.00	1.25 ±0.01	1.29 ±0.19	1.33 ±0.03
	2 [d]	1.40 ±0.04	1.49 ±0.00	1.73 ±0.00	1.68 ±0.07	1.82 ±0.02	2.03 ±0.03	1.77 ±0.01
	3 [d]	9.65 ±0.03	10.30 ±0.44	11.66 ±0.00	12.52 ±0.04	12.38 ±0.00	11.43 ±0.04	10.84 ±0.00
	4 [d]	1.48 ±0.07	1.52 ±0.09	1.87 ±0.00	1.88 ±0.00	1.71 ±0.00	1.72 ±0.06	1.67 ±0.00
	5 [d]	5.82 ±0.18	6.38 ±0.19	7.09 ±0.00	7.63 ±0.00	7.43 ±0.00	7.50 ±0.19	7.26 ±0.00
	6 [d]	5.70 ±0.19	6.15 ±0.20	7.44 ±0.00	7.24 ±0.02	7.27 ±0.01	7.29 ±0.16	7.21 ±0.02
	7 [d]	2.41 ±0.04	0.89 ±0.06	0.97 ±0.00	0.96 ±0.00	1.05 ±0.00	1.19 ±0.04	1.11 ±0.01
	8 [d]	0.76 ±0.02	0.71 ±0.03	0.76 ±0.00	0.72 ±0.01	0.84 ±0.00	0.96 ±0.02	0.81 ±0.00
	9 [d]	2.57 ±0.09	3.31 ±0.11	3.21 ±0.00	3.32 ±0.00	3.64 ±0.00	4.09 ±0.21	3.55 ±0.02
	10 [d]	0.54 ±0.03	0.67 ±0.04	0.64 ±0.00	0.68 ±0.00	0.73 ±0.02	0.86 ±0.03	0.67 ±0.00
	11 [d]	0.17 ±0.01	0.20 ±0.00	0.18 ±0.01	0.20 ±0.00	0.20 ±0.00	0.22 ±0.00	0.19 ±0.01
	12 [d]	0.55 ±0.04	0.68 ±0.01	0.67 ±0.01	0.83 ±0.04	0.74 ±0.00	1.04 ±0.01	0.77 ±0.00

[a] Results are presented as means ± sd (n = 3), Content is the value of dry weight; [b] Temperature; [c] Moisture (%); [d] units: mg/g. Numbering of the compounds is the same as the Table S2-1.

Table S5. The analysis between oven drying method and moisture meter method.

	**Oven Drying Method**	**Moisture Meter Method**
Mean	62.82	61.87
Variance	1.72	0.41
Observed value	7	7
p (T ≤ t) 2-tailed	0.08	
t 2-tailed critical	2.18	

$p > 0.05$.
